# Diversity of culturable gut bacteria and their role in conferring resistance to alpha-cypermethrin in field populations of *Stegomyia aegypti*

**DOI:** 10.3389/fmicb.2026.1749347

**Published:** 2026-04-14

**Authors:** Mahima K Mani, Sankari Thirumal, Vijayakumar Balakrishnan, Rakesh Singh, I Geetha

**Affiliations:** 1 Department of Microbiology and Immunology, ICMR-Vector Control Research Centre, Puducherry, India; 2 Department of Clinical and Molecular Medicine, ICMR-Vector Control Research Centre, Puducherry, India; 3 Division of Biostatistics and VBD Modeling, ICMR-Vector Control Research Centre, Puducherry, India; 4 Department of Microbiology, Jawaharlal Institute of Postgraduate Medical Education and Research, Puducherry, India

**Keywords:** alpha-cypermethrin, detoxification, LT50 bioassay, MALDI-TOF MS, mosquito gut microbiota

## Abstract

**Introduction:**

Control of *Stegomyia aegypti* (*St. aegypti*), a major vector of arboviruses, has been increasingly challenged by insecticide resistance, driven by genetic, metabolic, behavioral, and environmental factors. While extensively studied in other contexts, the role of gut microbiota in insecticide resistance remains largely unexplored. Therefore, this study assessed the α-cypermethrin susceptibility status of *St. aegypti* from Puducherry by characterizing the culturable gut bacteria from resistant and susceptible mosquito populations and evaluating their role in mediating resistance.

**Materials and methods:**

Mosquito larvae were collected from Puducherry, India, reared, and emerged as female *St. aegypti*, which were tested for α-CP (0.05%) susceptibility following World Health Organisation (WHO) guidelines, along with laboratory strains as controls. Larval and adult guts were dissected, and culturable bacteria were isolated and identified using MALDI-TOF MS and 16S rRNA sequencing. The resistant populations were treated with vancomycin, gentamicin, and streptomycin to suppress the gut microbiota and reassessed for α-CP susceptibility. The gut bacterial load was quantified by qPCR, and bacterial isolates were evaluated for *in vitro* degradation ability in minimal salt medium containing α-CP.

**Results:**

The *St. aegypti* populations tested from three locations in Puducherry exhibited confirmed resistance to α-CP. A total of 35 gut bacterial isolates were obtained (α-CP-resistant −26 and α-CP-susceptible −9). Compared to susceptible mosquitoes, resistant populations showed greater gut bacterial diversity and higher bacterial load. The resistant mosquitoes were dominated by *Bacillota* (adults 46.6%, larvae 54.5%) and *Pseudomonadota* (adults 46.6%, larvae 45.4%), with *Bacillaceae* and *Enterobacteriaceae* being the most abundant. The antibiotic-mediated microbiota suppression increased mortality (vancomycin 97%, gentamicin 95%, streptomycin 92%) and reduced LT50 following α-CP exposure. Further, isolates such as *Enterobacter hormaechei* and *Bacillus* spp. demonstrated growth in α-CP supplemented minimal salt media.

**Discussion:**

These findings suggest a strong association between gut microbiota and α-CP resistance in *St. aegypti.* The higher microbial diversity, evenness, and bacterial load observed in resistant populations, along with increased mortality following antibiotic treatment and the ability of certain isolates to grow in α-CP–supplemented media, indicate a potential symbiont-mediated mechanism contributing to insecticide resistance.

## 1 Background

Mosquitoes are the most common vectors transmitting pathogens that cause diseases like chikungunya, dengue, Zika, malaria, and filariasis. Dengue, an arboviral disease, is prevalent in temperate countries, with around 390 million people infected annually (Bhatt et al., 2013). The World Health Organisation (WHO) reported a tenfold increase in reported cases worldwide between 2000 and 2019, rising from half a million to 5.2 million. Most of the arboviral diseases transmitted by mosquitoes have no vaccine, and treatment is limited to supportive care. Hence, vector control becomes the method of choice, and chemical insecticides are crucial in managing mosquito-borne diseases globally. A variety of insecticides are being used to target both larval and adult stages of the mosquito vector. However, the overuse and abuse of these insecticides have resulted in widespread development of resistance among the mosquito population (Wang et al., 2023).

Mosquitoes harbor symbiotic bacteria, and larvae acquire these microbes during the aquatic phase, eventually becoming part of the commensal gut microbiota (Gimonneau et al., 2014). *Asaia i*s the most prevalent and stable symbiotic bacterium that coexists with *An. stephensi* (Favia et al., 2007). Other bacterial genera that are prevalent in mosquitoes include *Pantoea* and *Acinetobacter.* It has been found that the mosquito midgut microbiota affects several factors, including larval growth, susceptibility to arboviral infection (Apte-Deshpande et al., 2012; Guégan et al., 2018; Minard et al., 2013), blood digestion, egg production, and longevity (Harrison et al., 2023).

Furthermore, recent studies have shown that insecticide resistance is associated with symbiotic gut bacteria (Käch et al., 2018; Xia et al., 2013). Emerging evidence reveals that gut microbiota may significantly modulate resistance phenotypes, with specific bacterial taxa enriched in mosquitoes surviving high concentrations of pyrethroids, organophosphates, and other commonly used insecticides (Barnard et al., 2019; Djondji Kamga et al., 2025; Käch et al., 2018; Viafara-Campo et al., 2025; Xia et al., 2013). The midgut microbiota mediate resistance in two ways: one is by directly metabolizing chemical insecticides (Kikuchi et al., 2012) and the other is by increasing the activity of detoxification enzymes or enhancing their gene expression (Pang et al., 2018).

In recent years, few studies have investigated the gut microbiome and its role in insecticide resistance across different mosquito species from various regions worldwide. Studies across *Stegomyia aegypti* (Viafara-Campo et al., 2025), *Anopheles gambiae*, *Anopheles funestus,* and *Anopheles arabiensis* (Djondji Kamga et al., 2025; Worku et al., 2025) have revealed that resistant mosquitoes often harbor distinct microbial profiles, and depletion of these bacteria through antibiotic treatment increases insecticide susceptibility. In two different species of *Anopheles* mosquitoes, the role of gut bacteria in mediating resistance to fenitrothion and temephos was reported (Dada et al., 2018; Soltani et al., 2017). Similarly, in *Culex pipiens* and *St. aegypti*, *Wolbachia* in the gut microbiota is positively correlated with the development of organophosphorus insecticide resistance by regulating the esterase gene (Hoffmann and Turelli, 2013).

*Stegomyia* species of mosquito are the major vectors that transmit dengue and chikungunya, and the role of gut microbiota in resistance development has been reported in different countries. In *St. albopictus,* the bacterium *Serratia marcescens* was shown to enhance deltamethrin resistance by upregulating detoxification enzymes and related genes, particularly in the midgut and malpighian tubules, highlighting its role in symbiont-mediated insecticide resistance (Deng et al., 2025). In the United States, a permethrin-selected strain of *Stegomyia aegypti* (*St. aegypti*) exhibited higher bacterial richness and distinct gut microbial composition with degradation of xylan, chitin, and chlorophenols, suggesting an association between gut bacteria and resistance (Muturi et al., 2021). Similarly, two studies from Colombia revealed substantial alteration in the gut bacterial load and its association with insecticide resistance, in which Lambda-cyhalothrin-resistant *St. aegypti* populations showed altered gut bacterial diversity and were linked to insecticide detoxification (Arévalo-Cortés et al., 2020, 2022). Another recent study in Colombia highlighted the role of *Serratia*, the dominant bacterium, in temephos-resistant larvae and deltamethrin-resistant females, while the role of *Cedecea* in resistant females was highlighted for the potential role of gut bacteria mediating insecticide tolerance and degradation (Viafara-Campo et al., 2025). In a study using 16S rRNA amplicon sequencing in field-collected *St. albopictus* from China, significant differences in gut microbial diversity and abundance between resistant and sensitive strains were demonstrated in resistant adults (Wang et al., 2021).

Thus, only a limited number of studies on *St. aegypti* are currently available regarding the role of gut microbiota in modulating insecticide resistance, despite its critical influence on various physiological processes. Alpha-cypermethrin (α-CP), a pyrethroid, is widely used to control the vector *St. aegypti* (Linnaeus) (Diptera: Culicidae). In India, no studies have directly investigated the relationship between gut bacterial communities and insecticide resistance. To address this knowledge gap, the present study investigated the association between gut microbiota and α-CP resistance by culturing the gut bacteria of wild *St. aegypti* mosquitoes collected from different locations in Puducherry.

## 2 Methods

### 2.1 Mosquito collection and rearing

**Figure 1:**
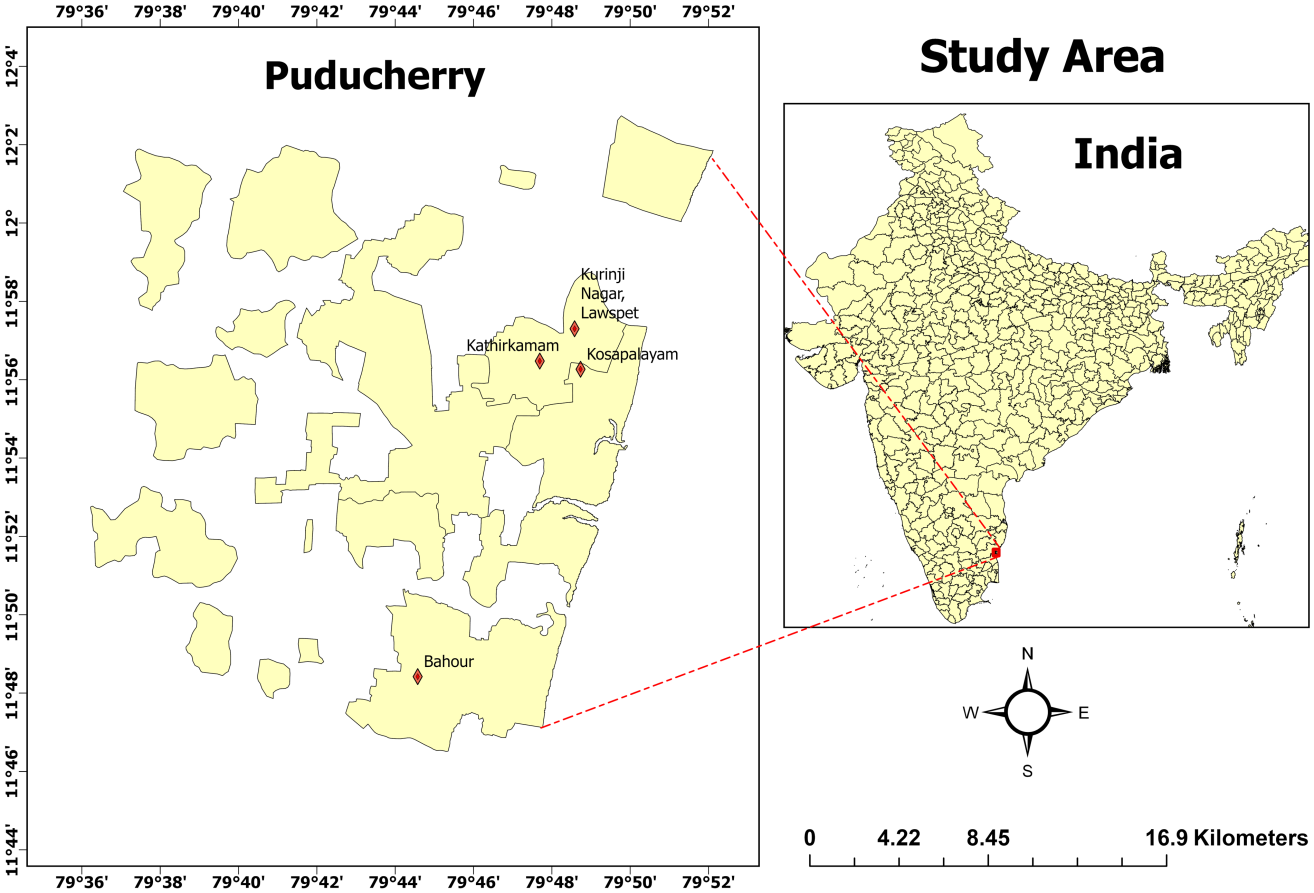
Mosquito collection areas in Puducherry. For further details, see in Supplementary Tables S1–S3.

Collections were carried out from March 2023 to August 2023, and the study sites were selected based on previous reports of pyrethroid usage and documented dengue incidence, indicating potential selection pressure for insecticide resistance. Mosquito larvae (third/fourth stage instar) were collected randomly from four sites in Puducherry Lawspet (11° 57′16.02655″N, 79° 48′23.75728″E), Kathirkamam (11° 57′1.46448″N, 79° 47′0.37″E), Kosapalayam (11° 56′6″N, 79° 48′50″E) and Bahour (11° 48′31″N, 79° 44′54″E) (Figure 1). Larvae were collected from breeding habitats (containers) surrounding the houses, and ovitraps were also placed in the same habitats for egg collection. The field-collected larvae were transferred to an enamel tray and maintained at 27 ± 2° C, 70–80% RH. Pupae were kept inside a BugDorm cage (32.5 cm × 32.5 cm × 32.5 cm), and emerged adults (F_1_) were fed 10% sterile sucrose. *St. aegypti* mosquitoes were identified using standard taxonomic keys (Rueda, 2004) and were used for testing insecticide susceptibility.

### 2.2 Susceptibility of *Stegomyia aegypti* to alpha-cypermethrin

The insecticide susceptibility testing of adult mosquitoes in the WHO tube test was carried out using α-CP (0.05%) impregnated papers (15 cm × 12 cm) obtained from the Vector Control Research Unit at University Sains Malaysia (USM). As recommended by the WHO, field-collected *St. aegypti* (F1 progeny) mosquitoes were used for susceptibility assays following the WHO guidelines (World Health Organization, 2022). 3–5-day-old, non-blood-fed female mosquitoes collected from three study sites (Lawspet, Kathirkamam, and Kosapalayam) were tested for α-CP susceptibility. For reference control, ICMR-VCRC insectary-reared *St. aegypti* females were used. For each location, 150 adult mosquitoes were used. Of these, 100 females were exposed to α-CP using WHO tube kits for 1 h, with four biological replicates per test (4 exposure tubes × 25 mosquitoes per tube), while 50 mosquitoes in two tubes served as unexposed controls to monitor natural mortality (Supplementary Figure S3). A total of 450 mosquitoes (300 test + 150 control) were used for the assay. After 1 h of exposure to α-CP in WHO-impregnated tubes, the mosquitoes were transferred back to the holding tubes and maintained under standard laboratory conditions for a 24-h recovery period. Mortality was then recorded after 24 h in accordance with WHO guidelines.

### 2.3 Isolation and identification of gut bacteria from α-CP susceptible and α-CP resistant *Stegomyia aegypti*

#### 2.3.1 Isolation

Fourth instar larvae (*n* = 50) and adult mosquitoes (3-day-old) (*n* = 50) were dissected to remove the gut for the isolation of bacteria from the α-CP resistant (α-CP-Res) and α-CP susceptible (α-CP-Sus) populations of *St. aegypti.* Briefly, the mosquitoes were immobilized by cold shock, followed by surface sterilization with sterile distilled water for 2 min, followed by immersion in 70% alcohol (molecular grade Hayman, UK) for 3 min, and then a final rinsing in sterile distilled water for 2 min (Soltani et al., 2017; Wang et al., 2022). Guts were dissected under a stereomicroscope (WESWOX® OPTIK SZM-102, India) in sterile normal saline (Supplementary Figures S1, S2), pooled (*n* = 10), and transferred to 100 μL Brain Heart Infusion (BHI) broth [Analytical grade, HiMedia, India]. Samples were homogenized (TissueLyser II, Qiagen) at 20 Hz for 10 min, then incubated at 37 °C for 16 h to enrich fastidious bacteria. Each group (α-CP-Res and α-CP-Sus) used five pools of 50 mosquito guts from larvae and adults. Following incubation, samples were diluted (10^−8^), plated on Tryptic Soy Agar (TSA) [Analytical grade, HiMedia, India], and incubated at 37 °C for 16 h. Distinct bacterial colonies were streaked on TSA, maintained on slants, and labeled as VCRCAR (adult-resistant), VCRCLR (larvae-resistant), VCRCAS (adult-susceptible), and VCRCLS (larvae-susceptible) (Supplementary Figures S4, S5).

#### 2.3.2 Identification of gut bacteria by matrix assisted laser desorption ionization–time of flight mass spectrophotometry

The isolated gut bacteria were identified by matrix assisted laser desorption ionization–time of flight mass spectrophotometry (MALDI-TOF MS) Biotyper (bioMérieux Vitek^®^). All isolates maintained on slants were subcultured onto TSA and NYSM plates, and after 18 h of incubation, colonies of each organism were smeared on the grids of the MALDI-TOF slide. The spotted bacterial sample was allowed to dry completely, and 1 μL of matrix solution (α-cyano-4-hydroxycinnamic acid) was added and mixed. The appropriate parameters, such as laser intensity, acquisition range, and the slide identification number, were assigned for the analysis, and the slides were loaded into the Biotyper. Using the VITEK MS system, the collected mass spectra were compared to the database’s spectra, and the bacteria were identified (Supplementary file S4).

#### 2.3.3 Gut bacteria identification by 16S rRNA sequencing

Bacterial isolates that could not be reliably identified by MALDI-TOF MS were further characterized by 16S rRNA gene sequencing for accurate taxonomic identification. Bacterial genomic DNA (gDNA) was extracted using the GenElute Bacterial Genomic DNA Kit (Sigma-Aldrich, United States), and the 16S rRNA gene was amplified using universal primers 8F 5′AGAGTTTGATCCTGGCTCAG3′ and 1942R 5′GGTACCTTGTTACGACTT3′ (Weisburg et al., 1991). The PCR reaction included 12.5 μL GoTaq® Green master mix (Promega Corporation), 0.5 μL of 10 pmol forward and reverse primers, 2 μL template DNA, and nuclease-free water to a final volume of 25 μL. The PCR cycling conditions included an initial denaturation at 94 °C for 3 min, followed by 35 cycles of denaturation at 94 °C for 1 min, annealing at 53 °C for 1 min, and extension at 72 °C for 1 min, followed by a final extension at 72 °C for 10 min. Amplicons were resolved on 1.5% agarose gel, purified using the Nucleospin Gel and PCR Cleanup Kit (Macherey-Nagel, Germany), and sequenced using the BigDye Terminator cycle sequencing kit V3.1 (Applied Biosystem, United States) as per the manufacturer’s instructions and purified by using the Nucleoseq purification column (Macherey-Nagel, Germany). Sequenced products were analyzed on a 3130 XL Genetic Analyzer, and chromatograms were processed with Chromas (v2.01). Forward and reverse reads were assembled into contigs using BioEdit (v7.2.6.1), and sequences were identified through BLAST search against the nucleotide database and the sequences were submitted to GenBank.

#### 2.3.4 Phylogenetic analysis

Four of the 16S rRNA sequences, which were identified by blast search against the nucleotide nr/nt database, were further subjected to phylogenetic analysis to confirm the taxonomic identification of the species. For the phylogenetic analysis, all four 16S rRNA sequences were individually blast searched against the GenBank nucleotide database. The sequences (one sequence per species with the highest similarity) that had a similarity of >98.7% with query sequences (threshold for differentiating species using the 16S rRNA gene) were retrieved (Kim et al., 2014). Multiple sequence alignment was performed using the MUSCLE algorithm, and the phylogenetic tree was constructed using maximum likelihood estimation based on the Tamura-Nei model with 500 bootstrap iterations in MEGA 11.0 software (Tamura et al., 2021). The outgroups were selected for each of the four 16S rRNA sequences in such a way that they belonged to the same family but to different genera.

### 2.4 Suppression of gut microbiota in α-CP-res *Stegomyia aegypti* by antibiotic treatment

To determine the role of gut flora in α-CP resistance, the gut flora was cleared from the resistant population by feeding them with the sucrose solution laced with antibiotics. For this, newly emerged adult female mosquitoes (F_1_) from the α-CP-Res population were released into four cages, each consisting of 200 female mosquitoes. Each group was fed streptomycin [Molecular grade, HiMedia India], gentamicin [Molecular grade, HiMedia India], and vancomycin [Molecular grade, HiMedia India] from the first day of post-eclosion (Arévalo-Cortés et al., 2020; Gómez-Govea et al., 2022; Martinson and Strand, 2021; Qing et al., 2020). Antibiotics were administered at 50 μg/mL, a concentration selected based on previous studies, to ensure broad-spectrum activity against both Gram-positive and Gram-negative bacteria while effectively suppressing gut microbiota without significant toxicity to mosquitoes. After 3 days of antibiotic treatment, the mosquitoes were tested for susceptibility to α-CP (0.05%) by the WHO tube test as described earlier. The susceptibility test was carried out with four populations, viz., α-CP-Res (control), α-CP-Res treated with streptomycin, α-CP-Res treated with gentamicin, and α-CP-Res treated with vancomycin (Supplementary Figure S7). The lethal time (LT50) was also determined by recording the mortality every 5 min during a 60 min exposure, and the percentage mortality was calculated after 24 h.

### 2.5 *In vitro* degradation ability of α-CP by the gut bacteria

The ability of *in vitro* degradation of α-CP by the gut bacteria isolated from both the population was tested by culturing them in M9 Minimal salt medium [Analytical grade, HiMedia, India] (disodium hydrogen phosphate −6.78 g/L, potassium dihydrogen phosphate- 3.0 g/L, sodium chloride- 0.5 g/L, ammonium chloride- 1.0 g/L) supplemented with 15 mM α-CP (Technical grade, S. R. chemicals and pharmaceuticals, India). Two sets of M9 Minimal salt medium plates were prepared, each set containing 0.1 and 0.05% added glucose [Analytical grade, Himedia, India] as a carbon source. Initially, all the gut bacterial isolates were streaked onto media containing 0.1% of glucose and incubated at 37 °C for up to 96 h. The bacterial isolates that showed growth were further plated on media supplemented with 0.05% glucose, incubated as above, and observed for the growth of bacteria.

### 2.6 Quantification of gut bacteria in α-CP-Res, α-CP-Sus, and vancomycin-treated field populations of *Stegomyia aegypti* by real-time PCR

The total gut bacteria in α-CP-Res *St. aegypti* emerged from field-collected larvae, and vancomycin-treated field caught α-CP-Res *St. aegypti* was quantified in comparison with insectary-reared α-CP-Sus population by qPCR targeting the 16S rRNA gene. The vancomycin-treated group was selected for quantification because it exhibited the least survival upon exposure to α-CP. Genomic DNA was extracted from mosquito gut homogenate consisting of 30 mosquito guts in normal saline from each of the three groups using the DNeasy blood and tissue kit (Qiagen, Germany) according to the manufacturer’s instructions. Quantification of gut bacteria was performed using the universal 16S rRNA primers by amplifying 16S rRNA fragments as reported by Wei et al. (2017) in the CFX96™ Real-Time PCR Detection System (Bio-Rad, United States). The *St. aegypti* ribosomal protein S17 (*RPS17*) housekeeping gene was used as an endogenous control. The reaction mix consisted of 7 pmol each of the forward and reverse primers of the 16S rRNA gene, 7 pmol each of the forward and reverse primers of the *RPS17* gene, 10 μL of iQ SYBR green super mix (BioRad, United States), and 20 ng of DNA, and the reaction volume was adjusted to 20 μL using nuclease-free water. Optimized PCR conditions include an initial denaturation at 95 °C for 3 min, 40 cycles of denaturation at 95 °C for 15 s, annealing at 60 °C for 40 s, and melting curve analysis at 55 °C for 10 s and 95 °C for 5 s.

### 2.7 Statistical analysis

The Shannon, Simpson, and Pielou’s evenness indices were calculated to analyze bacterial diversity of the gut flora in the α-CP-Res and α-CP-Sus populations of *St. aegypti.* The survival rate of each mosquito after antibiotic treatment and the LT50 values were estimated by Kaplan–Meier Survival analysis using IBM® SPSS® Statistics_28.0. The survival curves for each antibiotic-treated and control groups were compared by the Log–Rank test. The effect of antibiotic treatment on gut bacterial load by qPCR was analyzed using a non-parametric Mann–Whitney test to find the differences in gut bacterial load in the antibiotic-treated and control groups. In all tests, the significance level was set at *p* < 0.05.

## 3 Results

### 3.1 Mosquito collection and rearing

A total of 3,253 *Stegomyia* larvae were collected from four different locations in Puducherry, India, of which 3,212 *Stegomyia* have emerged as adults (≥98%). Furthermore, 914 *St. aegypti* mosquitoes emerged from eggs collected through ovitraps placed at the same study sites. More than 50% (51.79%) larvae were collected from the area Kathirkamam, Puducherry (Supplementary Tables S1, S2). The majority of the emerged adults were identified as *St. aegypti* (*n* = 3,150), and 57% of the emerged adults were females. The House index, Container index and Breteau index were calculated for all the four sites and found to be high in Kathirkamam, as compared to other collection areas. The majority of the collection sites showed entomological indices above threshold levels (HI ≤ 1%, BI ≤ 4%), indicating the possibility of dengue transmission in these areas (Supplementary Table S3).

### 3.2 Susceptibility of *Stegomyia aegypti* to alpha-cypermethrin

**Figure 2:**
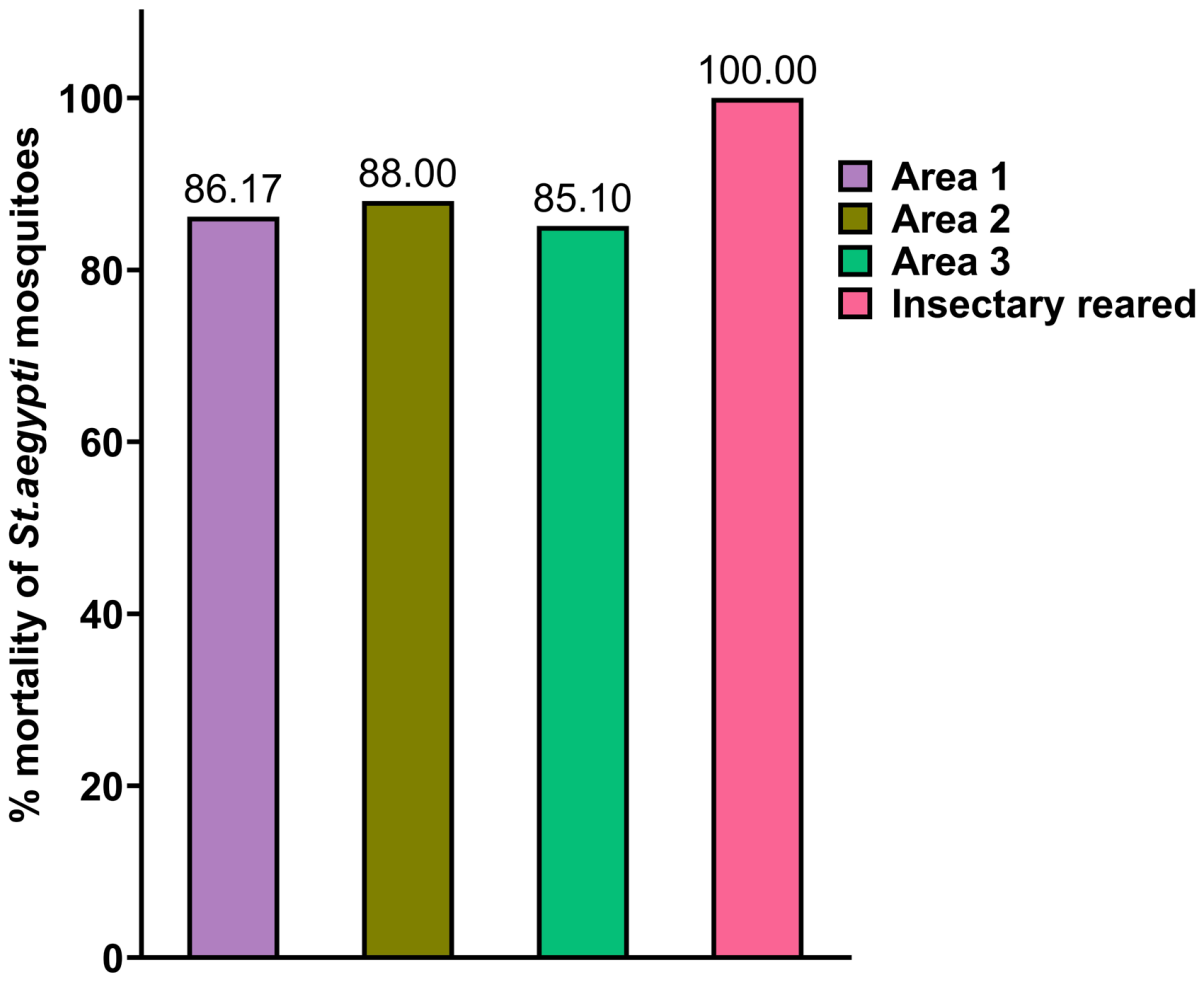
The susceptibility status of field-collected and insectary-reared *St. aegypti* against alphacypermethrin (0.05%). The *St. aegypti* collected from three areas showed percentage mortality below 90%, indicating confirmed resistance to α-CP (0.05%) according to the WHO tube test protocol. The insectary-reared mosquitoes showed 100% mortality (susceptibility) to α-CP (0.05%).

According to WHO guidelines, for tube tests, the mosquito mortality rate of ≥98% indicates susceptibility to the tested insecticide; while ≥90% but <98% indicates possible resistance, and <90% indicates confirmed resistance. The results of the WHO tube test for α-CP susceptibility showed that *St. aegypti* collected from three different areas in Puducherry were resistant to α-CP (0.05%) (Figure 2). Kathirkamam (area 3) showed the highest resistance level with a mortality rate of 85.1% on α-CP exposure, followed by Lawspet-86.17% (area 1) and Kosapalayam −88% (area 2). The VCRC insectary-reared *St. aegypti* colony was found to be susceptible (100% mortality) to α-CP (Supplementary Tables S4–S7).

### 3.3 Isolation and identification of gut bacteria from α-CP susceptible and α-CP resistant *Stegomyia aegypti*

**Figure 3:**
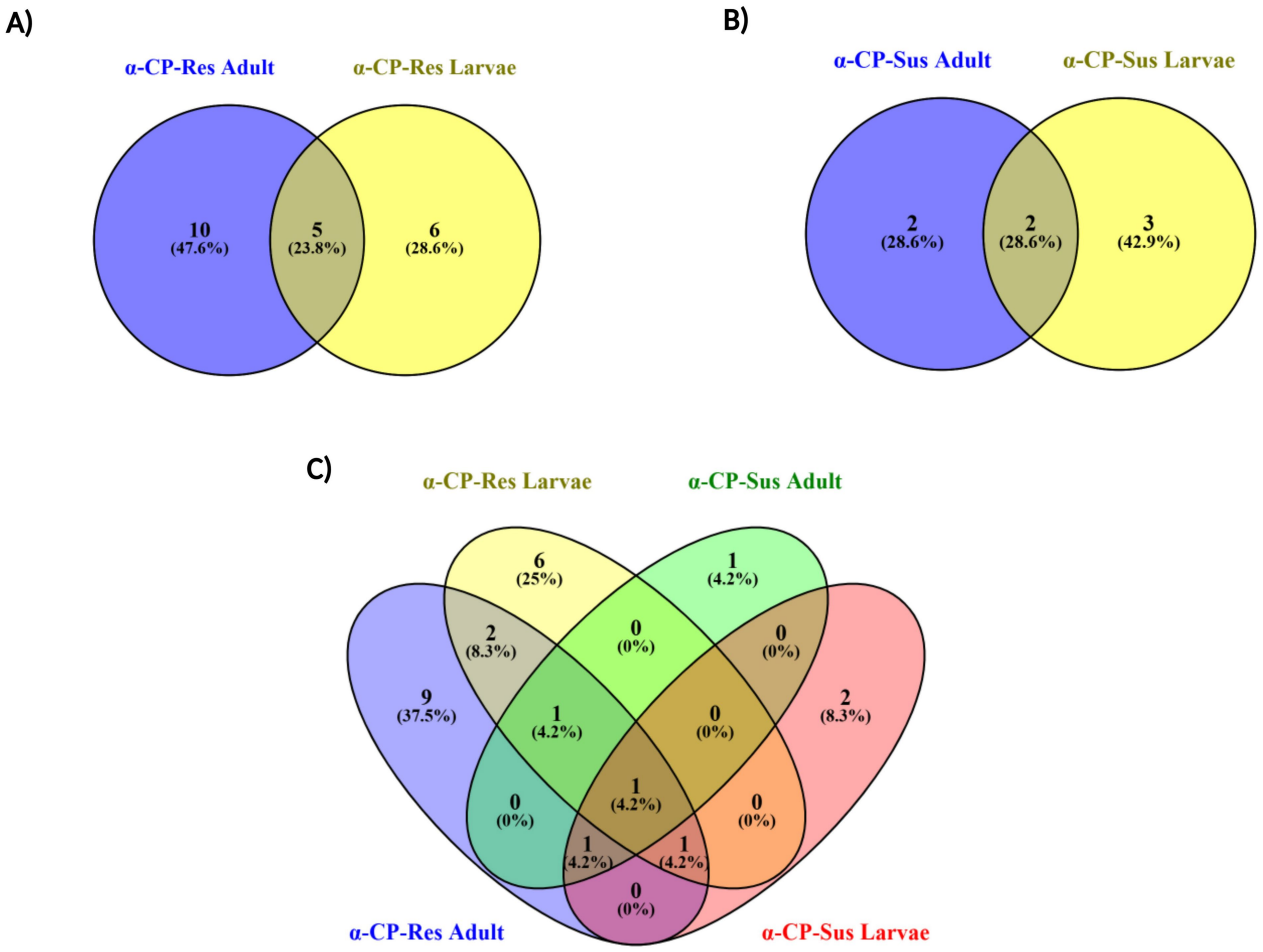
Venn diagram showing distribution of bacterial isolates between α-CP-Res and α-CP-Sus populations of *Stegomyia aegypti*. The Venn diagram illustrates the number of unique and shared bacterial isolates identified from the **(A)** α-CP-Res adult and larval gut and **(B)** α-CP-Sus adult and larval gut. **(C)** Comparative distribution of bacterial isolates identified from the gut of α-CP-Res adult, α-CP-Res larvae, α-CP-Sus adult, and α-CP-Sus larvae. Numbers in non-overlapping regions represent isolates unique to each life cycle stage and resistance status, while overlapping regions indicate bacterial isolates common between adults and larvae within resistant or susceptible populations. This comparison highlights differences in gut bacterial composition associated with insecticide resistance and developmental stage.

A total of 35 bacteria were isolated from the gut of α-CP-Res (*n* = 26) and α-CP-Sus (*n* = 9) *St. aegypti*. From the gut of larvae with α-CP-resistance, 11 bacteria were isolated, while, from the adult gut 15 bacteria were isolated. In the case of α-CP-Sus *St. aegypti,* 4 bacteria from the adult gut and five bacteria from the larval gut were isolated. Among the 35 isolates, 18 were identified as unique while the remaining 17 isolates shared across different life stages of both populations (Figure 3).

Of these 35 isolates, 31 were successfully identified by MALDI-TOF MS based on acquired mass spectra, while 4 isolates that could not be resolved by MALDI-TOF MS were subsequently identified by 16S rRNA sequencing (Table 1). These four isolates failed to revive from glycerol stocks, and due to the lack of fresh, viable cultures required for protein profiling, definitive identification by MALDI-TOF MS was not possible.

**Table 1:** Bacteria identified by MALDI-TOF MS & 16S rRNA sequencing, isolated from the gut of α-CP-Res and α-CP-Sus population of *St. aegypti.*

Sample ID	Life stage and insecticide susceptibility status	Bacteria identified	Identification method
VCRCAR1	α-CP-Res Adult	*Bacillus cereus group*	MALDI-TOF MS
VCRCAR2	α-CP-Res Adult	*Klebsiella pnuemoniae*	MALDI-TOF MS
VCRCAR3	α-CP-Res Adult	*Chryseobacterium gleum*	MALDI-TOF MS
VCRCAR4	α-CP-Res Adult	*Lysinibacillus fusiformis*	MALDI-TOF MS
VCRCAR5	α-CP-Res Adult	*Enterococcus faecalis*	MALDI-TOF MS
VCRCAR6	α-CP-Res Adult	*Bacillus flexus*	MALDI-TOF MS
VCRCAR7	α-CP-Res Adult	*Enterobacter species*	MALDI-TOF MS
VCRCAR8	α-CP-Res Adult	*Staphylococcus aureus*	MALDI-TOF MS
VCRCAR9	α-CP-Res Adult	*Enterobacter hormaechei*	MALDI-TOF MS
VCRCAR10	α-CP-Res Adult	*B.subtilis/amyloliquefaciens/valismortis*	MALDI-TOF MS
VCRCAR11	α-CP-Res Adult	*Aeromonas hydrophilia*	MALDI-TOF MS
VCRCAR12	α-CP-Res Adult	*Pseudomonas aeruginosa*	MALDI-TOF MS
VCRCAR13	α-CP-Res Adult	*Escherichia coli*	MALDI-TOF MS
VCRCAR14	α-CP-Res Adult	*Enterococcus faecium*	MALDI-TOF MS
VCRCAR15	α-CP-Res Adult	*Acinetobacter baumannii* (PP535071)	16S rRNA sequencing
VCRCLR1	α-CP-Res Larva	*Bacillus cereus group*	MALDI-TOF MS
VCRCLR2	α-CP-Res Larva	*Staphylococcus epidermidis*	MALDI-TOF MS
VCRCLR3	α-CP-Res Larva	*Enterobacter cloacae*	MALDI-TOF MS
VCRCLR4	α-CP-Res Larva	*Aeromonas hydrophilia*	MALDI-TOF MS
VCRCLR5	α-CP-Res Larva	*Aeromonas punctata*	MALDI-TOF MS
VCRCLR6	α-CP-Res Larva	*Enterobacter asburiae*	MALDI-TOF MS
VCRCLR7	α-CP-Res Larva	*Staphylococcus aureus*	MALDI-TOF MS
VCRCLR8	α-CP-Res Larva	*Bacillus altitudinis/pumilus*	MALDI-TOF MS
VCRCLR9	α-CP-Res Larva	*Lysinibacillus fusiformis*	MALDI-TOF MS
VCRCLR10	α-CP-Res Larva	*Bacillus subtilis/amyloliquefaciens/valismortis*	MALDI-TOF MS
VCRCLR11	α-CP-Res Larva	*Shewanella decolorationis* (PP535072)	16S rRNA sequencing
VCRCAS1	α-CP-Sus Adult	*Enterobacter mori strain* (PP535073)	16S rRNA sequencing
VCRCAS2	α-CP-Sus Adult	*Bacillus subtilis/amyloliquefaciens/valismortis*	MALDI-TOF MS
VCRCAS3	α-CP-Sus Adult	*Bacillus cereus group*	MALDI-TOF MS
VCRCAS4	α-CP-Sus Adult	*Klebsiella pneumonia*	MALDI-TOF MS
VCRCLS1	α-CP-Sus Larva	*Staphylococcus aureus*	MALDI-TOF MS
VCRCLS2	α-CP-Sus Larva	*Lactococcus garvieae*	MALDI-TOF MS
VCRCLS3	α-CP-Sus Larva	*Bacillus cereus group*	MALDI-TOF MS
VCRCLS4	α-CP-Sus Larva	*Klebsiella pneumonia*	MALDI-TOF MS
VCRCLS5	α-CP-Sus *Larva*	*Microbacterium* sp. (PP535074)	16S rRNA sequencing

The phylogenetic tree constructed showed that each of the four 16S rRNA sequences was found to cluster with *Acinetobacter baumannii, Shewanella decolorationis, Enterobacter mori,* and *Microbacterium* sp., respectively (Supplementary file 3).

#### 3.3.1 The diversity of gut bacteria in field-collected α-CP-res *Stegomyia aegypti*

**Figure 4:**
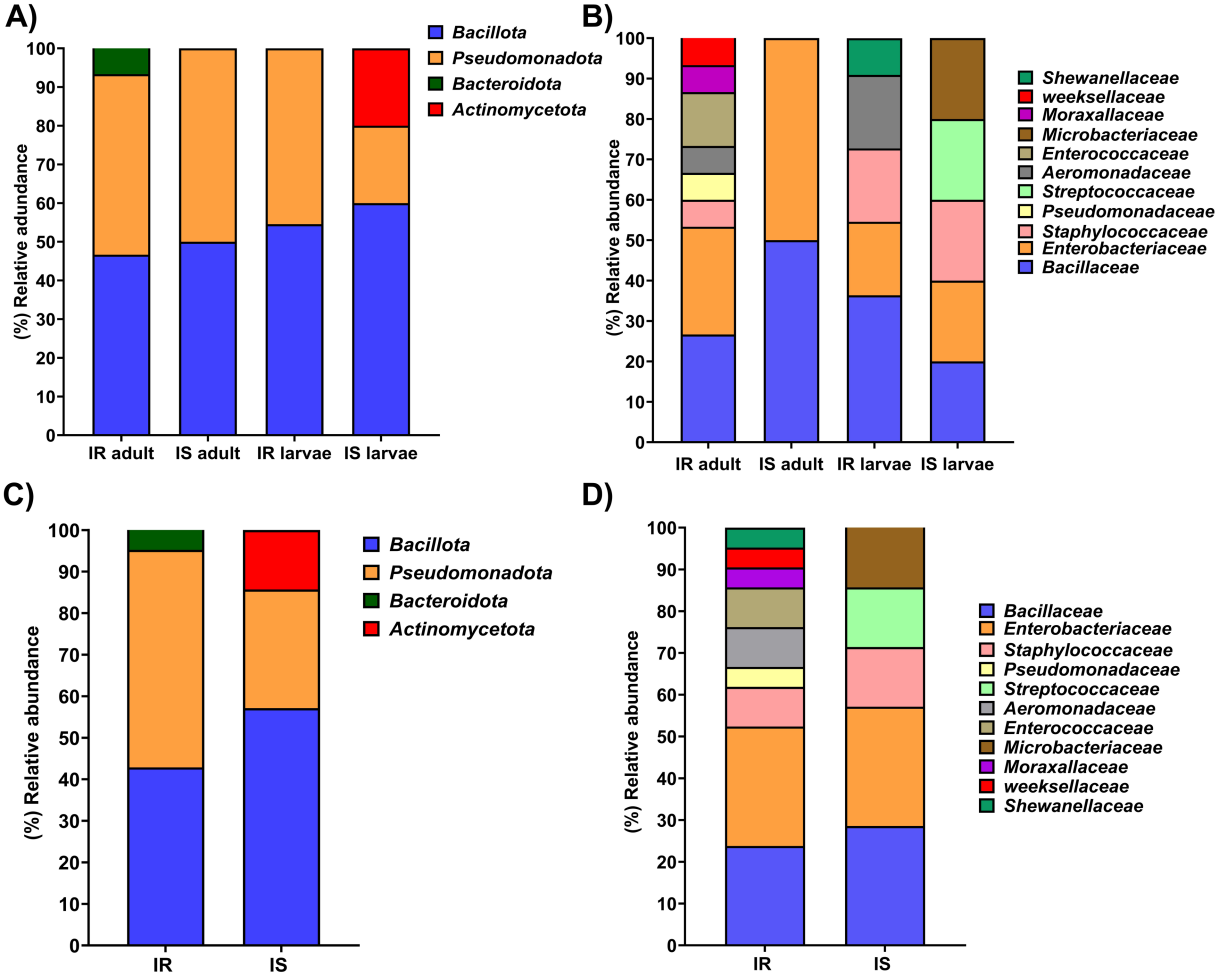
Relative abundance of bacterial isolates. Relative abundance of bacterial isolates at **(A)** phyla level **(B)** family level identified from the larval and adult gut of α-CP-Res and α-CP-Sus *St. aegypti*. The overall abundance of bacterial isolates at the phyla **(C)** and family level **(D)** from the α-CP resistant (IR) and susceptible (IS) population of *St. aegypti*.

A total of 26 bacterial species were identified from the larval and adult gut of the α-CP-Res population of *St. aegypti* (adult-15, larvae-11). At the phylum level, the microbiota was predominantly composed of 13 isolates from *Bacillota (firmicutes)*, which contributed to 50% of the total bacterial species identified. Of the 15 isolates from adult gut, 7 (46.6%) species belonged to *Bacillota*, while 6 of the 11 larval isolates (54.5%) fell under the same phylum (Figure 4A). The second most predominant phylum was *Pseudomonadota (Proteobacteria),* comprising 12 species, which is 46.1% of the total bacterial isolates identified in α-CP-Res *St. aegypti*. In the adult gut, seven species (46.6%) were assigned to *Pseudomonadota*, and 5 species (45.4%) from the larval gut also belonged to the same phylum. Out of the 26 isolates, 25 isolates belong to the phylum *Bacillota* and *Pseudomonadota,* and the remaining one species belonged to the phylum *Bacteroidota* (3.84%), which was also detected in the adult gut (Figures 4A,C).

In the adult α-CP-Res population, bacteria belonging to eight different families were identified (Figure 4B). The most dominant families were *Bacillaceae* and *Enterobacteriaceae* (each accounting for 26.7% of the total adult gut microbiota). Other families include *Staphylococcaceae* (6.7%), *Pseudomonadaceae* (6.7%), *Aeromonadaceae* (6.7%), *Enterococcaceae* (13.3%), *Moraxellaceae* (6.6%), and *Weeksellaceae* (6.7%) (Figure 4B).

In resistant larvae, five bacterial families were detected (Figure 4B). *Bacillaceae* was the most abundant (36.36%), followed by *Enterobacteriaceae* (18.1%), *Staphylococcaceae* (18.1%), *Aeromonadaceae* (18.1%), and *Shewanelleaceae* (9.0%). When considering both life stages (adult and larval), the α-CP-resistant population of *St. aegypti* harbored mainly bacteria belonging to the families *Enterobacteriaceae* (28.57%) and *Bacillaceae* (23.80%) (Figure 4D).

#### 3.3.2 The diversity of gut bacteria in insectary collected α-CP-Sus *Stegomyia aegypti*

In the α-CP-Sus population, a total of nine bacterial species were identified, of which 4 were isolated from adults and 5 from larvae. At the phylum level, *Bacillota* was the dominant phylum comprising five species (55.6%), followed by *Pseudomonadota* with three species (33.3%). One species, belonging to *Actinomycetota* (11.1%), was detected exclusively in larvae (Figure 4C). In adult mosquitoes, both the phyla, *Bacillota* and *Pseudomonadota,* were equally represented (50% each). While in larvae, *Bacillota* was the most abundant (60%) phylum, followed by *Pseudomonadota* and *Actnimycetota* (20% each) (Figure 4C).

The adult mosquitoes harbored bacteria belonging to families *Bacillaceae* and *Enterobacteriaceae*, each contributing to 50% of the total bacteria identified. However, in larvae, five bacterial families were present, which include *Enterobacteriaceae*, *Staphylococcaceae*, *Aeromonadaceae*, *Microbacteriaceae,* and *Bacillaceae* in equal abundance (20.0%) (Figure 4B). Overall, in the gut of the α-CP-Sus population of *St. aegypti,* bacterial species belonging to the families *Bacillaceae* and *Enterobacteriaceae* were equally abundant (28.5%) (Figure 4D). Furthermore, species belonging to *Staphylococcaceae* (14.3%), *Streptococcaceae* (14.3%), and *Microbacteriaceae* (14.3%) were also detected (Figure 4D).

**Figure 5:**
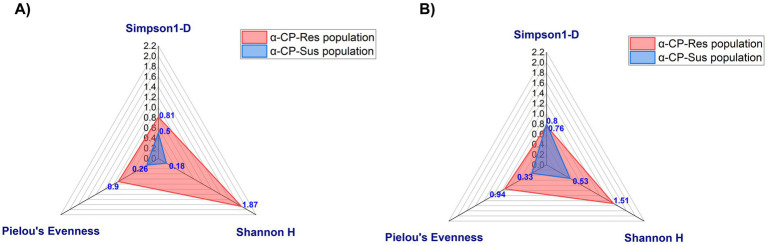
Alpha diversity of gut microbiota isolated from **(A)** adult and **(B)** larvae of α-CP-Res and α-CP-Sus populations. Simpson 1-D, Shannon H, and evenness of gut microbiota from α-CP-Res population and α-CP-Sus populations isolated from **(A)** adult and **(B)** larvae.

The microbial diversity of the gut flora of α-CP-Sus and α-CP-Res populations of *St. aegypti* was analyzed by calculating three alpha diversity indices, Simpson 1-D, Shannon H index, and Pielou’s evenness index (Figure 5). In the larvae, the Simpson 1-D diversity index is quite similar between the resistant (0.76) and susceptible (0.8) populations. However, the Shannon diversity index is notably higher in resistant larvae (1.51) than in susceptible larvae (0.53), indicating a more diverse gut microbiota. Evenness, which reflects the distribution of species, is also much higher in resistant larvae (0.94) than in susceptible larvae (0.33), suggesting a more balanced microbial community in resistant individuals.

The resistant adult population maintains higher diversity, with a Simpson index of 0.81 and a Shannon index of 1.87, compared to the susceptible adults, which have values of 0.5 and 0.18, respectively. Evenness remains high in resistant adults (0.9) but is considerably lower in susceptible adults (0.26). These findings suggest that the resistant population harbors a richer and more evenly distributed microbial community in both larvae and adult stages.

### 3.4 Suppression of gut microbiota in α-CP-Res *Stegomyia aegypti* by antibiotic treatment

**Figure 6:**
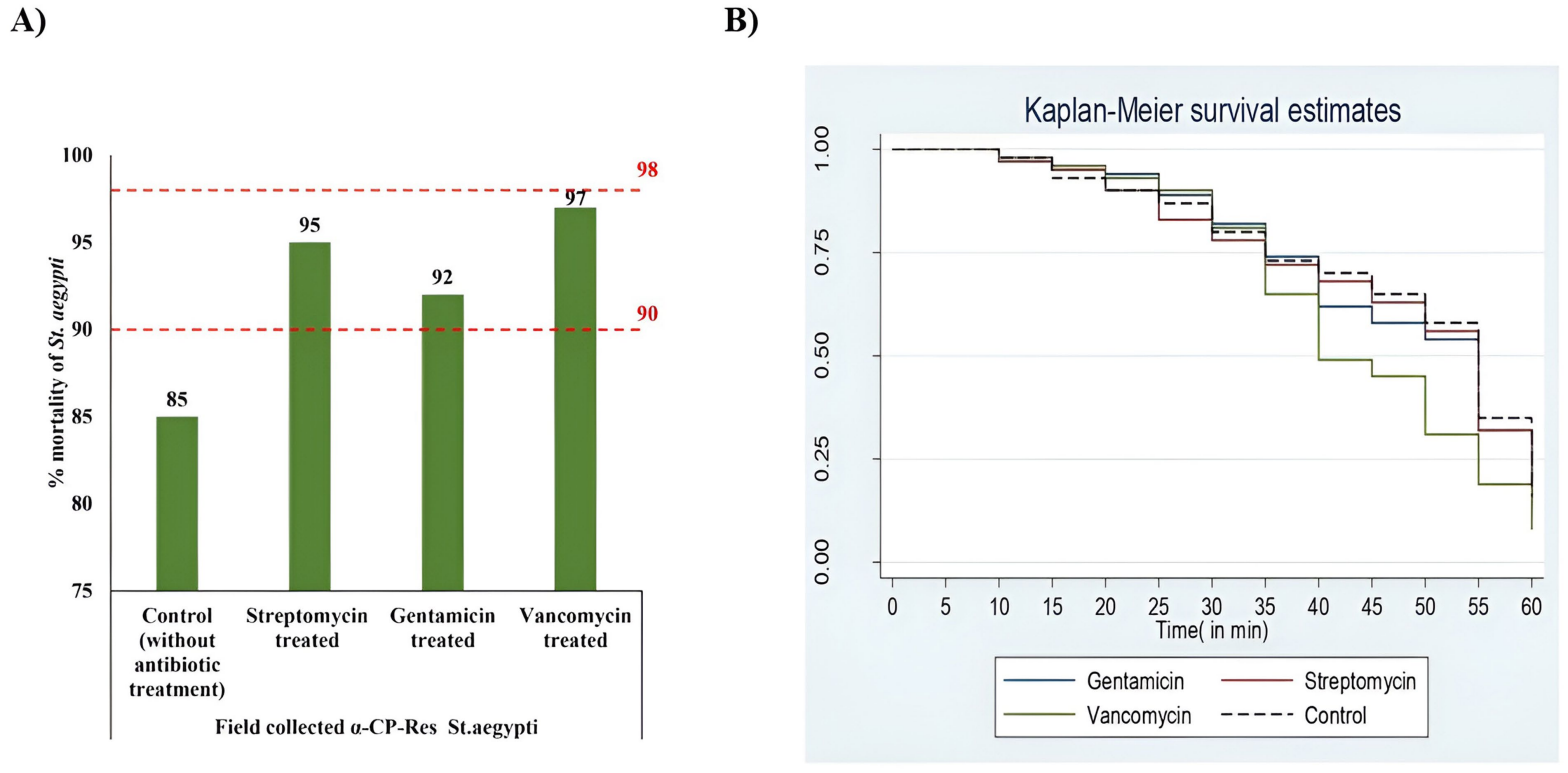
Effect of antibiotic treatment on α-CP—susceptibility and survival in field-collected *St. aegypti.* **(A)** Changes in susceptibility status of field-collected *St. aegypti* to α-CP (0.05%) following antibiotic treatment. The control group consisted of initially confirmed α-CP-resistant mosquitoes without antibiotic exposure (intact gut microbiota). Antibiotic-treated groups included streptomycin-, gentamicin-, and vancomycin-treated mosquitoes. Upon exposure to α-CP (0.05%) using the WHO tube test, antibiotic-treated groups exhibited increased mortality rates (>90%) compared to the control group (85%). According to WHO criteria, mortality between 90 and 98% indicates possible resistance, whereas mortality ≤90% confirms resistance. Thus, the untreated control group remained resistant, while the antibiotic-treated groups shifted toward possible resistance status. **(B)** Kaplan–Meier survival curves of antibiotic-treated and untreated (control) *St. aegypti* exposed to α-CP (0.05%). Survival analysis followed by the Log-Rank test revealed statistically significant differences among groups (χ^2^ = 10.010, df = 3, *p* = 0.0119). The control group (without antibiotics) showed the highest survival following insecticide exposure. Streptomycin and gentamicin-treated groups exhibited survival patterns comparable to the control, with an estimated LT50 of 55 min. Conversely, the vancomycin-treated group demonstrated the lowest survival and a reduced LT50 of 40 min. These findings indicate that antibiotic-mediated disruption of gut microbiota reduced the tolerance of *St. aegypti* to lethal concentrations of α-CP.

To study the suppression of gut microbiota, field-collected *St. aegypti* were fed streptomycin, gentamicin, and vancomycin. During the antibiotic feeding period, no mortality was observed. However, when exposed to α-CP, the antibiotic-treated mosquitoes exhibited higher mortality than the untreated group. Among the three antibiotic groups, the vancomycin-treated group had the highest mortality rate, at 97%, followed by the gentamicin-treated group with 95%, and the streptomycin-treated group at 92%. Conversely, the untreated field-collected population was α-CP resistant, as they showed 85% mortality rate. After antibiotic treatment to clear the gut flora, these populations exhibited a “possible resistance” status, with mortality rates between 92 and 97% (Figure 6).

### 3.5 *In vitro* degradation of α-CP by gut bacterial isolates

Among the 35 gut bacteria tested for degradation of α-CP, only three bacterial isolates from the α-CP-Res population of *St. aegypti* showed growth on M9 Minimal salt media. *Bacillus altitudinis/pumilus* isolated from the adult gut and *B. subtilis/amyloliquefaciens* and *Enterobacter hormachei* from the larval gut were able to degrade α-CP (Supplementary Figure S8). These three bacteria showed that they utilize α-CP as a carbon source and can grow on the medium containing only 0.05% of glucose. None of the isolates isolated from the α-CP-Sus population of *St. aegypti* in the present study were able to degrade the insecticide.

### 3.6 Quantification of gut bacteria in α-CP-Res, α-CP-Sus, and vancomycin-treated field population of *Stegomyia aegypti* by real-time PCR

**Figure 7:**
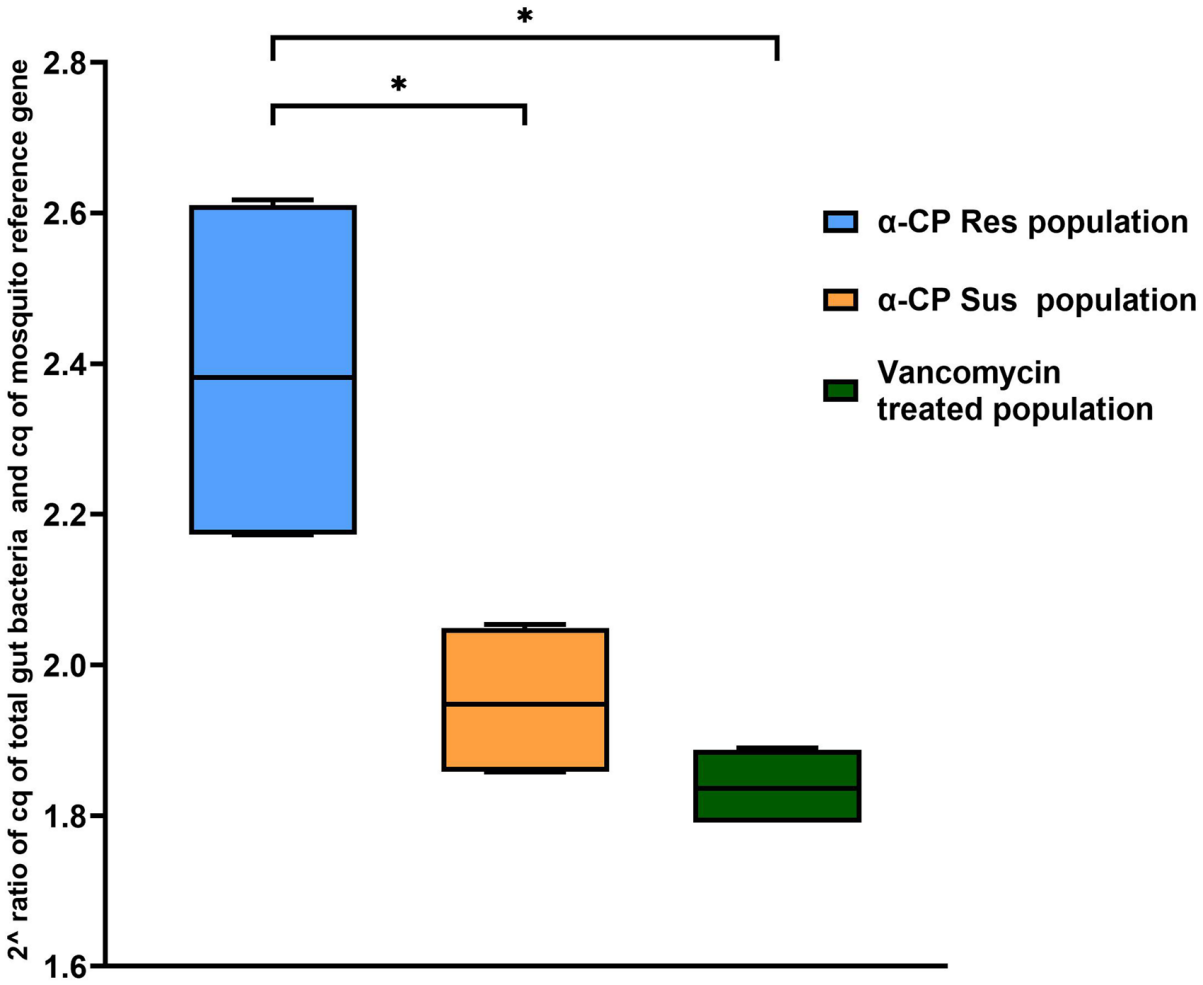
Quantification of gut bacteria. Total gut bacteria quantified in the field caught alphacypermethrin-resistant, vancomycin-treated, and insectary-reared alphacypermethrin susceptible populations of *St. aegypti* by qPCR. *p < 0.05.

The α-CP-Res population showed the highest quantity of gut bacterial flora, followed by the α-CP-Sus population as determined by qPCR. Vancomycin-treated α-CP-Res *St. aegypti* exhibited a significant reduction in gut bacterial load (Figure 7). The differences in the 2^ ratio of Cq values between the 16S rRNA gene of total gut bacteria and the mosquito reference gene across the three groups were analyzed using the Mann–Whitney test. The results showed a significant difference in gut bacterial abundance between the α-CP-Res and α-CP-Sus populations, with the field-collected α-CP-Res group having a higher bacterial load than the α-CP-Sus group (*p* = 0.030). After antibiotic treatment, the gut bacterial load in the α-CP-Res population significantly decreased (*p* = 0.030). However, there was no significant difference in the bacterial load between the vancomycin-treated α-CP-Res group and the insectary-reared α-CP-Sus population (*p* = 0.31). These findings suggest that antibiotic treatment effectively reduced gut flora abundance, bringing the bacterial load in the α-CP-Res population closer to that of the insectary-reared α-CP-Sus group.

## 4 Discussion

The connection between gut microbiota and insecticide resistance is largely underexplored, with limited data on the role of microbial diversity in insecticide-resistant mosquitoes (Apte-Deshpande et al., 2012; Barnard et al., 2019; Dada et al., 2018; Gómez-Govea et al., 2022; Muturi et al., 2021; Wang et al., 2022). The present study highlights how variations in the gut flora of *St. aegypti* may influence resistance mechanisms, suggesting that microbiota composition could be a major factor in modulating insecticide susceptibility.

Larval collections and entomological indices indicate that three study sites except Bahour, (area 4) Puducherry, India, exceeded the threshold levels, signaling an increased risk of dengue outbreaks and the urgent need for vector control interventions. The high prevalence of *St. aegypti* (79%) further underscores the importance of focused surveillance and control efforts to reduce dengue transmission.

The *St. aegypti* mosquito population in Puducherry has recently been reported to show resistance to pyrethroid deltamethrin (Sadanandane et al., 2022). Furthermore, accumulating data from numerous Indian studies indicates growing resistance to both pyrethroids and organophosphates (Jangir and Prasad, 2022). In our study, all the field-collected *St. aegypti* were found to be resistant to α-CP (0.05%), while the laboratory-reared *St. aegypti* exhibited susceptibility. This disparity can be attributed to the lack of insecticide exposure and stable environmental conditions in the laboratory over multiple generations. Conversely, field populations continuously exposed to insecticides are likely to develop and maintain resistance mechanisms, possibly mediated by both genetic and microbial factors. Moreover, the detection of widespread resistance in all the collection areas suggests an urgent call for the management of insecticide resistance in dengue-endemic areas.

Two studies have reported the successful use of MALDI-TOF MS for bacterial identification in mosquito guts (Tandina et al., 2018; Gazzoni Araújo Gonçalves et al., 2019). In our study, of the 35 samples processed, 31 isolates were identified at the species level, while the remaining four samples, which failed in MALDI-TOF, were analyzed by constructing a phylogenetic tree using 16S rRNA sequencing and identified as *Enterobacter mori., Acinetobacter baumanni*, *Microbacterium* sp., and *Shewanella decolorationis* sp. Although the MALDI-TOF MS method is robust for bacterial identification, it is less effective in distinguishing between closely related *Bacillus* species, such as the *B. cereus* group. In our study, MALDI-TOF has identified the bacteria that are resistant to α-CP as *B. altitudinis/pumilus* and *B. subtilis/amyloliquefaciens/valismortis*. Similar results were also reported by the 16S rRNA sequencing method, as the members of the *B. cereus* group have a sequence identity of 99 to 100% (Ash et al., 1991). Nonetheless, MALDI-TOF MS has proven to be a reliable molecular tool for the rapid identification of bacteria at the genus level in mosquito gut flora (Tandina et al., 2018; Gazzoni Araújo Gonçalves et al., 2019). This warrants the use of alternative taxonomic marker genes such as *rpoB* and *gyrA* protein-coding genes.

The microbial abundance was higher in the field-collected, adult and larval α-CP-Res population than the insectary population, in accordance with the results of previous studies (Baltar et al., 2023). The gut of insecticide-resistant mosquito population, that emerged from field-collected larvae, contained a higher number of morphologically unique colonies, indicating greater diversity. The Simpson’s 1-D, Shannon, and Pielou’s Evenness indices were higher in the resistant population, indicating greater species abundance and evenness in the gut bacterial flora of resistant *St. aegypti*. Field populations that encounter frequent insecticide exposure are likely to acquire and maintain a more diverse and specialized gut microbiome that helps them cope with insecticidal pressure. The lower diversity in laboratory-reared strains, maintained without any insecticide exposure, further substantiates the influence of environmental factors on microbial composition. Predominant bacterial genera, such as *Bacillus*, *Enterobacter*, and *Klebsiella*, were present in both populations.

The predominance of *Enterobateriaceae* in both resistant and susceptible populations indicates its essential role in the gut microbial community, while the presence of *Bacillaceae* and *Pseudomonadaceae* in resistant populations suggests potential adaptations that could be associated with the resistance phenotype (Qiao et al., 2023). In the present study, a dye-decolorizing bacterium *Shewanella decolorationis* was isolated and identified by 16S rRNA sequencing, representing the first report of this bacterium in the gut of *St. aegypti*. Some pathogenic bacteria such as *E. coli, Klebsiella pneumonia, Staphylococcus aureus, Pseudomonas aeruginosa* and *Acinetobacter baumannii* were also isolated from the gut of *St. aegypti.* The presence of bacteria like *K. pneumoniae* and *A. baumannii,* known for their antibiotic resistance, raises concerns about the potential spread of antibiotic resistance genes (Mondal et al., 2019; Rahman et al., 2018). The observed variation in bacterial diversity between field-collected and laboratory-reared *St. aegypti* suggests that environmental exposure and habitat-specific factors significantly shape the gut microbiome (Minard et al., 2013).

Our investigation regarding the possible role of gut bacteria to α-CP resistance indicates an outline of symbiont mediated resistance. Suppressing the gut flora of field-collected α-CP-resistant *St. aegypti* with antibiotics caused significant changes in insecticide susceptibility with vancomycin-treated groups showed statistically significant differences in the survival and LT50 compared to untreated controls. We could not find any significant differences in the survival and LT50 of streptomycin and gentamicin-treated groups. These findings align with previous studies indicating that gut bacteria can influence insecticide susceptibility (Gómez-Govea et al., 2022; Wang et al., 2022). Our results also demonstrate that gut bacteria might provide a protective role against insecticide-induced stress, possibly through the degradation of toxic compounds or modulation of the mosquito’s detoxification system.

The quantitative PCR results also reveal that the total bacterial load was reduced significantly (*p* = 0.030) after antibiotic treatment, and the population thus became more vulnerable to death due to alphacypermethrin. Antibiotic treatment might have resulted in the dysbiosis of gut bacteria which is indicated by the reduction in the total gut bacterial load thereby increasing the susceptibility to α-CP. This is evidenced by the percentage mortalities and LT50 values following insecticide exposure (Figure 6).

The ability of gut bacteria to degrade insecticides can play a crucial role in determining the survival and adaptation of mosquito populations, especially in areas of high insecticide use. Previous studies have demonstrated that insecticide resistance reduces the bacterial diversity by enriching the insecticide-degrading bacteria in the gut (Muturi et al., 2021; Wang et al., 2022). In the present study, three bacterial species, *Enterobacter hormaechei*, *Bacillus altitudinis/pumilus*, and *Bacillus subtilis/amyloliquefaciens,* were able to grow in the presence of alphacypermethrin, indicating their potential role in insecticide degradation.

The genus *Enterobacter* has been extensively studied for its ability to metabolize various xenobiotics, including organophosphates like chlorpyrifos and persistent organic pollutants such as polychlorinated biphenyls, and has been found to degrade pyrethroid residues (Jia et al., 2008; Liao et al., 2009; Singh et al., 2004). Their capacity to survive and proliferate in media containing α-CP in this study further supports the hypothesis that these bacteria might help mosquitoes cope with insecticide exposure by metabolizing or detoxifying these compounds. The presence of such bacteria in the gut could therefore influence the overall tolerance of mosquito populations to pyrethroid insecticides.

Similarly, species of the *Bacillus* genus, such as *Bacillus thuringiensis* and *Bacillus subtilis*, have been reported to degrade pyrethroids, with *Bacillus subtilis* specifically known for its involvement in the biodegradation of cypermethrin (Bhatt et al., 2020; Xiao et al., 2015). The ability of *B. altitudinis/pumilus* and *B. subtilis/amyloliquefaciens* to grow in the presence of α-CP in the current study aligns with the previous findings, indicating that they possess enzymatic pathways capable of breaking down pyrethroid molecules. Such bacteria could potentially reduce the toxic effects of pyrethroids on the host mosquitoes, thereby contributing to an increased survival rate in environments where these insecticides are widely used.

Notably*, Bacillus* sp. (*B. subtilus/B. amyloliquefaciens/B. valismortis*) were detected in both α-CP-Res larvae and adult and detoxified the insecticide. However, *B. subtilus/B. amyloliquefaciens/B. valismortis* present in α-CP-Sus adult did not exhibit insecticide degradation. This strongly indicates that these bacteria might have been acquired from larvae to adult through transstadial transmission, survived metamorphosis, and adapted to changes in adult diet and gut environment (Kumar et al., 2010; Moll et al., 2001; Oliveira et al., 2011; Short et al., 2017). The insecticide detoxification ability of *B. subtilis* can be due to its symbiotic relationship with other gut bacteria, which is evidenced by the high gut microbial diversity in both life cycle stages of α-CP-Res *St. aegypti* in comparison to the α-CP-Sus life stages (Kikuchi et al., 2012). *E. hormaechei* and *B. pumilus* are the other two insecticide -degrading bacteria detected, among that *E. hormaechei* was found exclusively in the α-CP-Res adult gut, but not in the larval gut, which can be explained by the fact that this bacterium would have occurred in low densities and not at the detectable level as a colony, while it might have thrived in the stable adult gut environment. Similarly, *B. pumilus* was unique to the α-CP-Res larval gut, while its absence in the adult gut can be attributed to its inability to survive the metamorphosis process, particularly during the pupal stage (Moll et al., 2001). However, future studies are warranted to confirm insecticide degradation through detailed metabolite profiling using gas chromatography mass spectrometry (GC–MS).

Our findings highlight important public health implications for insecticide resistance management. Gut microbiota may influence insecticide susceptibility in *St. aegypti*, and targeted manipulation of resistance-associated bacterial communities could enhance insecticide efficacy and restore susceptibility. Microbiome-based approaches, including microbial inhibitors or paratransgenesis, may complement existing control strategies, while gut bacterial profiles could serve as potential biomarkers for resistance surveillance. Furthermore, bioremediation using insecticide-degrading gut bacteria offers a promising, eco-friendly approach for detoxifying contaminated environments and minimizing residual chemical pollutants. However, the potential selection of insecticide-tolerant or degrading bacteria under continuous insecticide pressure underscores the need for careful implementation within integrated resistance management frameworks.

Limitations of the study: In this study, the cultivable gut microbiota of the insecticide-resistant and susceptible *St. aegypti* mosquito population were compared to isolate and confirm the *in vitro* insecticide-degrading ability of bacterial isolates. However, the key limitation of the study is the lack of 16S rRNA gene-based metagenome sequencing, which would have provided a comprehensive profile of gut microbiota in resistant and susceptible mosquito populations, as >99% of microbes are uncultivable. Such an approach would have enabled a more comprehensive understanding of microbial community composition, relative abundance, frequency, and functional pathways associated with insecticide resistance. Another important limitation of the study is that gut bacterial load was quantified only in adults, not in other life- cycle stages such as larvae and pupae. This would have led to the identification of larval-acquired bacteria and their maintenance or enrichment in the adult gut to understand the shift in gut flora and their survival during metamorphosis, adaptation to diet, and insecticide degradation.

## 5 Conclusion

The present study highlights the significant differences in gut microbial communities between insecticide-resistant and susceptible populations of *St. aegypti* and suggests a potential role of these microbiotas in modulating resistance. The bacterial phyla, *Bacillota* and *Proteobacteria*, dominated the gut of *St. aegypti* and explained why, in the absence of gut flora, when exposed to insecticide, the insecticide-resistant mosquitoes showed increased survival and mortality. Furthermore, the field population of *St. aegypti* harbors insecticide-degrading bacteria. Future research elucidating the exact biochemical pathways will provide insight into the exact mechanism by which these bacteria degrade pyrethroids. The findings of this study may provide a deeper understanding of the relationship between gut microbiota and insecticide resistance, paving the way for new avenues for insecticide resistance management. Monitoring changes in the gut microbiota may provide an early warning system for resistance development. Further, bioremediation using insecticide-degrading gut bacteria may serve as an eco-friendly solution for detoxifying contaminated environments and reducing chemical residues.

## Data Availability

The datasets presented in this study can be found in online repositories. The names of the repository/repositories and accession number(s) can be found in the article/Supplementary material.
