# Supplementary material for: Diversity of culturable gut bacteria and their role in conferring resistance to alpha-cypermethrin in field populations of Stegomyia aegypti
Source: Front Microbiol. 2026 Apr 14;17:1749347. doi: 10.3389/fmicb.2026.1749347 (PMC13121122; doi:10.3389/fmicb.2026.1749347)
Supplement: Supplementary file 1 [file Data_Sheet_1.PDF]

Supplementary table 1 -Site-Wise St. aegypti Larval Collection Data

Sl.no	Area	Total no. of Larvae	Total no. of adult emerged	No. of *St. aegypti*	
1	Lawspet	958	950	950	512 ♀<br>438 ♂
2	Kathirkamam	1685	1662	1600	927♀<br>673♂
3	Bahour	150	148	148	80 ♀<br>68 ♂
4	Kosapalayam	460	452	452	235 ♀<br>217 ♂
Total		3,253	3,212	3150	

Supplementary Table 2- Data St. aegypti emerged from eggs collected using ovitrap

Sl.no	Name of area	No. of Ovitraps	No.of eggs collected and floated	No. of female *St.aegypti* emerged
1	Kurinji Nagar, Lawspet	15	567	364
2	kathirkamam	10	419	200
3	Bahour	15	120	50
Total		40	1,106	914

Supplementary Table 3- Data on Larval Indices

Sl. No	Name of area	House index (HI)	Container index (CI)	Breteau index (BI)
1	Lawspet	10%	22.2%	13.3%
2	Kathirkamam	16.6%	40%.	20%
3	Bahour	0	0	0
4	Kosapalayam	13.3%	33.3%	13.3%

Supplementary Table 4 - Data on percentage mortality of St. aegypti collected from Kurinji Nagar, Lawspet, following exposure to Alphacypermethrin (0.05%)

Replicate number	Number Exposed	Number knocked down after 60 minutes of exposure (KD)	% KD	Mortality (moribund+dead) after 24 hours	% Treated mortality	% Control mortality
**T_1**	25	21	84	22	88	
**T_2**	25	21	84	21	84	
**T_3**	25	21	84	22	88	
**T_4**	25	22	88	22	88	
**C_1**	25	1	4	2		8
**C_2**	25	0	0	1		4
**Corrected mortality (%)**				**86.17**		

Supplementary Table 5- Data on percentage mortality of St. aegypti collected from Kosapalayam, following exposure to Alphacypermethrin (0.05%)

Replicate number	Number Exposed	Number knocked down after 60 minutes of exposure (KD)	% KD	Mortality (moribund+dead) after 24 hours	% Treated mortality	% Control mortality
**T_1**	25	23	92	23	92	
**T_2**	25	23	92	24	96	
**T_3**	25	22	88	22	88	
**T_4**	25	20	80	20	80	
**C_1**	25	1	4	2		8
**C_2**	25	0	0	2		4
**Corrected mortality (%)**				**88**		

Supplementary Table 6- Data on percentage mortality of St. aegypti collected from Kathirkamam, following exposure to Alphacypermethrin (0.05%)

Replicate number	Number Exposed	Number knocked down after 60 minutes of exposure (KD)	% KD	Mortality (moribund+dead) after 24 hours	% Treated mortality	% Control mortality
**T_1**	25	22	84	23	88	
**T_2**	25	20	84	20	84	
**T_3**	25	22	84	22	88	
**T_4**	25	21	88	21	88	
**C_1**	25	1	4	2		8
**C_2**	25	0	0	1		4
**Corrected mortality (%)**				**85.10**		

Supplementary Table 7-Data on percentage mortality of St. aegypti collected from VCRC insectary following exposure to Alphacypermethrin (0.05%).

Replicate number	Number Exposed	Number knocked down after 60 minutes of exposure (KD)	% KD	Mortality (moribund+dead) after 24 hours	% Treated mortality	% Control mortality
**T_1**	25	24	96	25	100	
**T_2**	25	25	100	25	100	
**T_3**	25	25	100	25	100	
**T_4**	25	25	100	25	100	
**C_1**	25	0	0	0		0
**C_2**	25	0	0	0		0
**Treatment mortality (%)**				**100**		

$$\text{Treatment mortality (\%)} = \frac{\text{Number of treated female mosquitoes dead}}{\text{Total number of treated female mosquitoes}} \times 100$$

$$\text{Control mortality (\%)} = \frac{\text{Number of control female mosquitoes dead}}{\text{Total number of control female mosquitoes}} \times 100$$

$$\text{Corrected mortality} = \frac{(\text{\% treated mortality - \% control mortality})}{(100 - \text{\% control mortality})} \times 100$$
