# Supplementary material for: Diversity of culturable gut bacteria and their role in conferring resistance to alpha-cypermethrin in field populations of Stegomyia aegypti
Source: Front Microbiol. 2026 Apr 14;17:1749347. doi: 10.3389/fmicb.2026.1749347 (PMC13121122; doi:10.3389/fmicb.2026.1749347)
Supplement: Supplementary file 2 [file Data_Sheet_2.PDF]

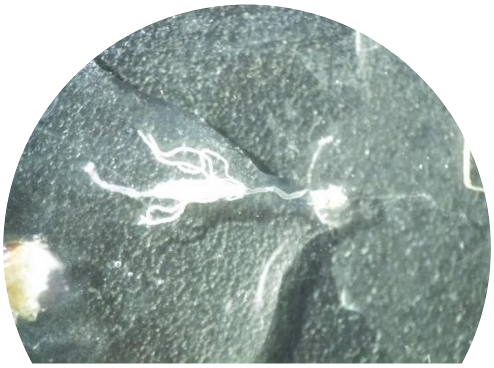

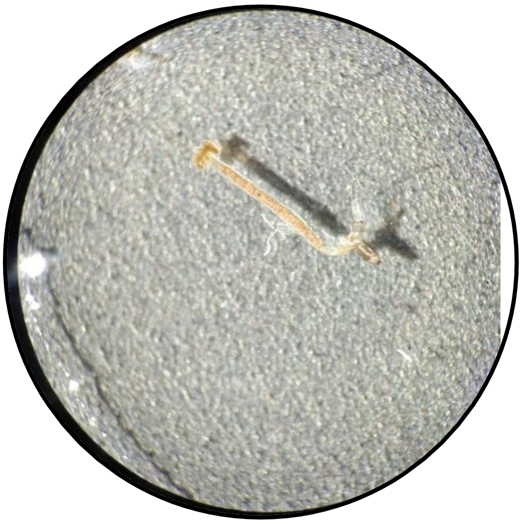

Supplementary Fig 1 - Gut of adult mosquito under dissection microscope

Supplementary Fig 2 - Gut of adult mosquito under dissection microscope

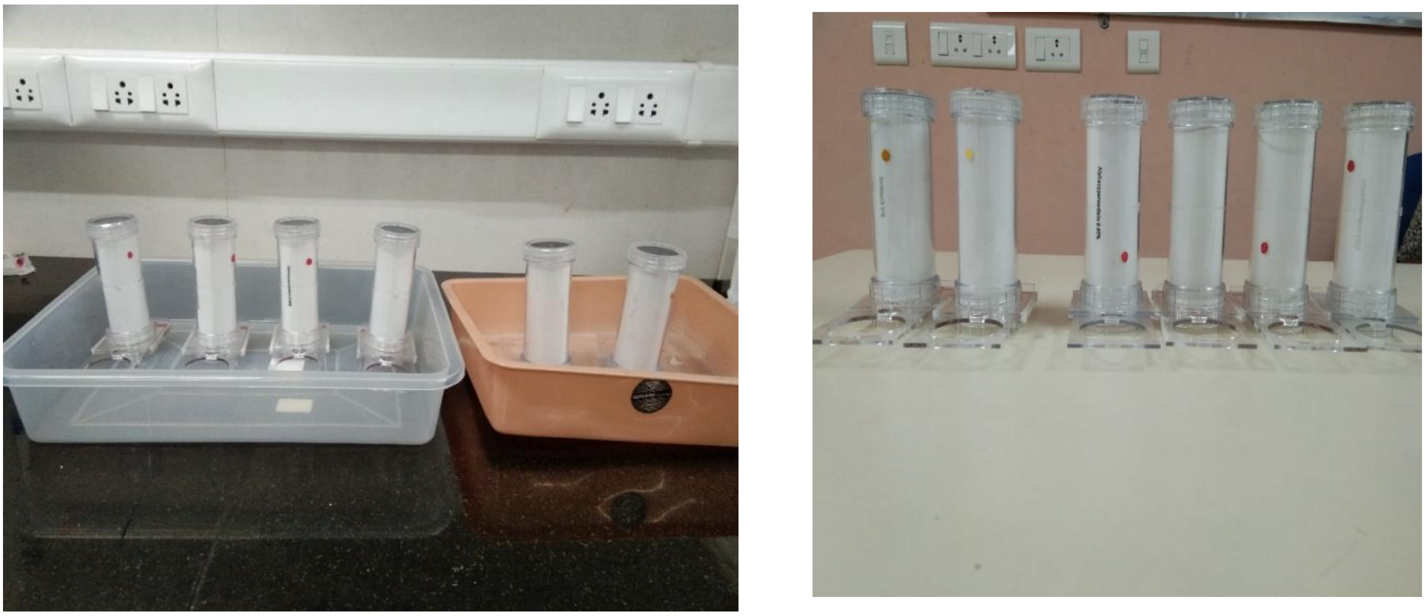

Supplementary Fig 3 - WHO tube test for α-cypermethrin susceptibility in field-collected St. aegypti mosquitoes

Supplementary Fig 4- Some of the morphologically distinct colonies obtained from the gut sample of St. aegypti

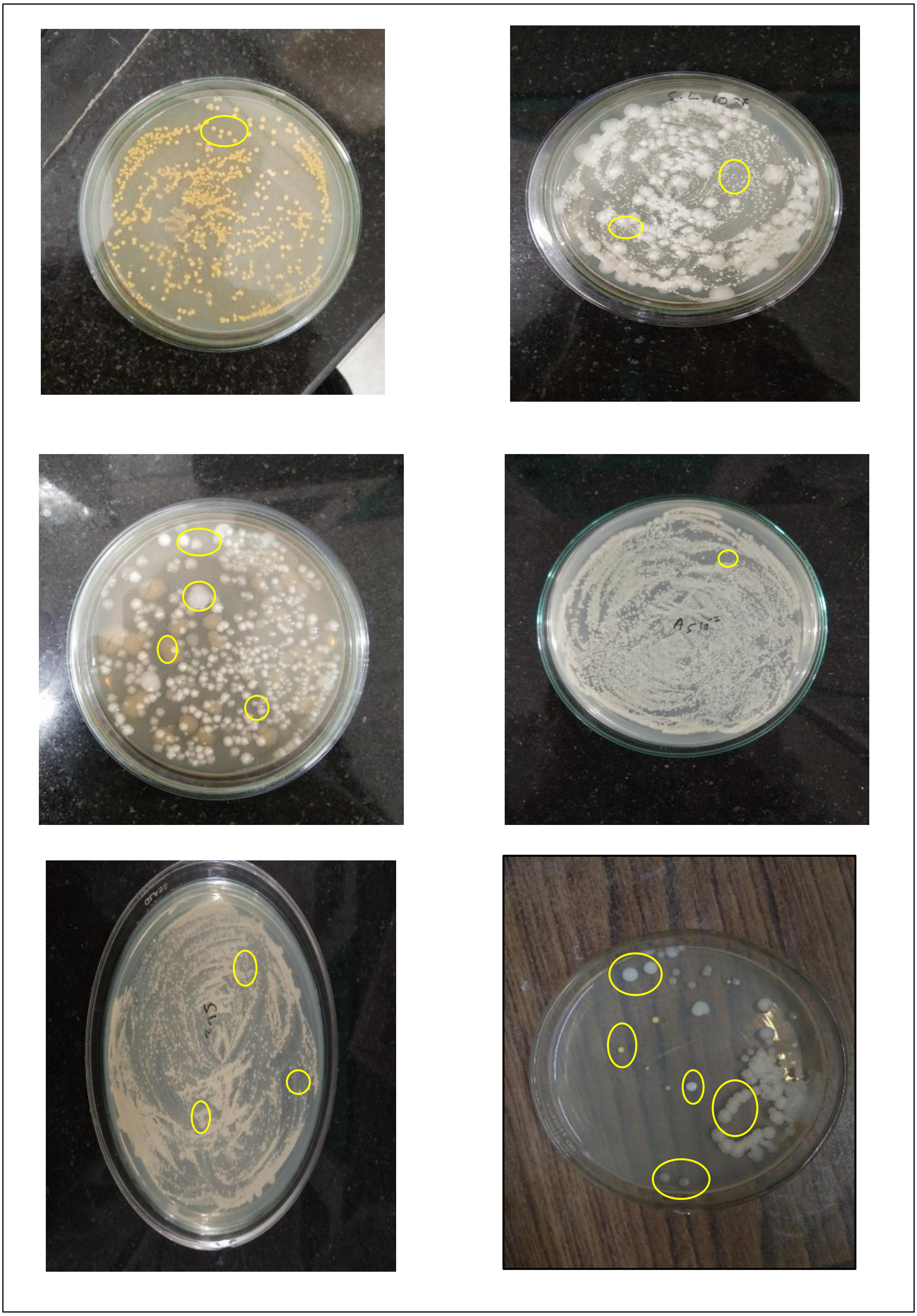

Supplementary Fig 5 -TSA plates showing pure colonies obtained from the gut sample

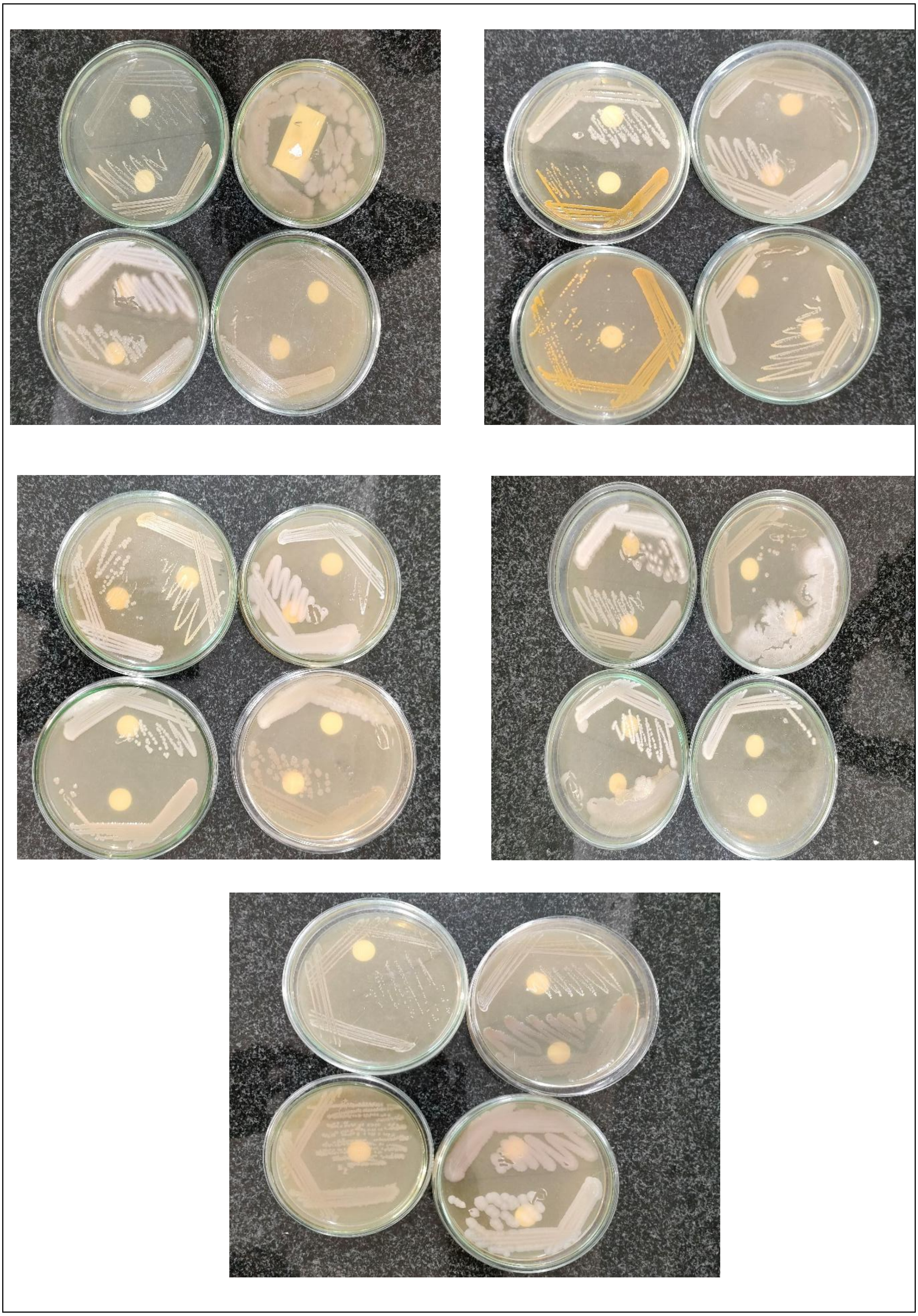

Supplementary Fig 7- WHO tube test of antibiotic treated St.aegypti mosquitoes

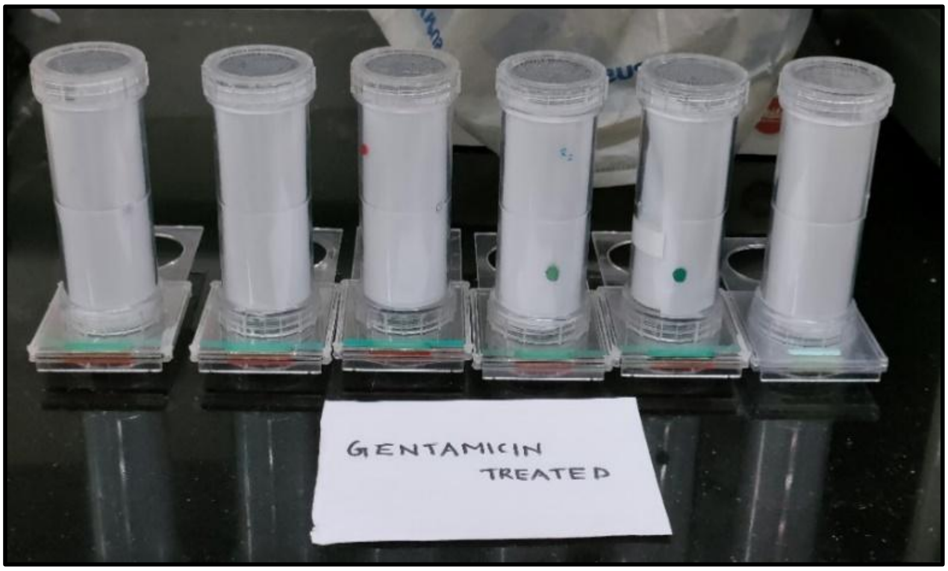

WHO tube test with Gentamicin treated population

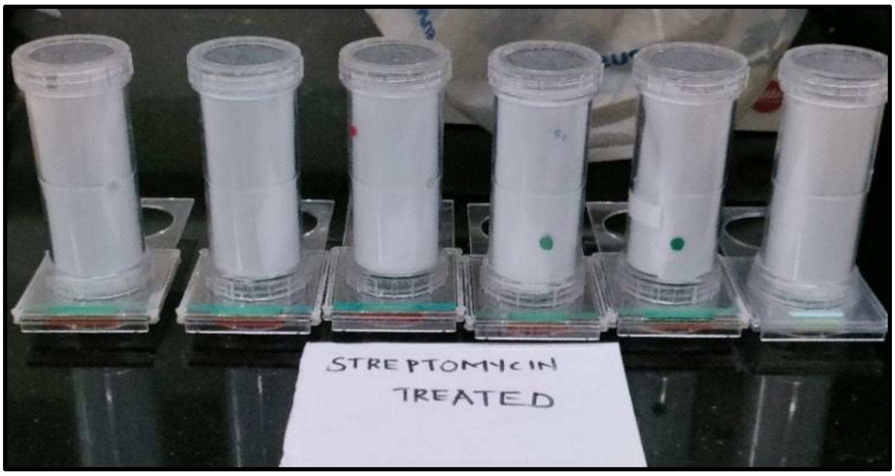

WHO tube test with Streptomycin treated population

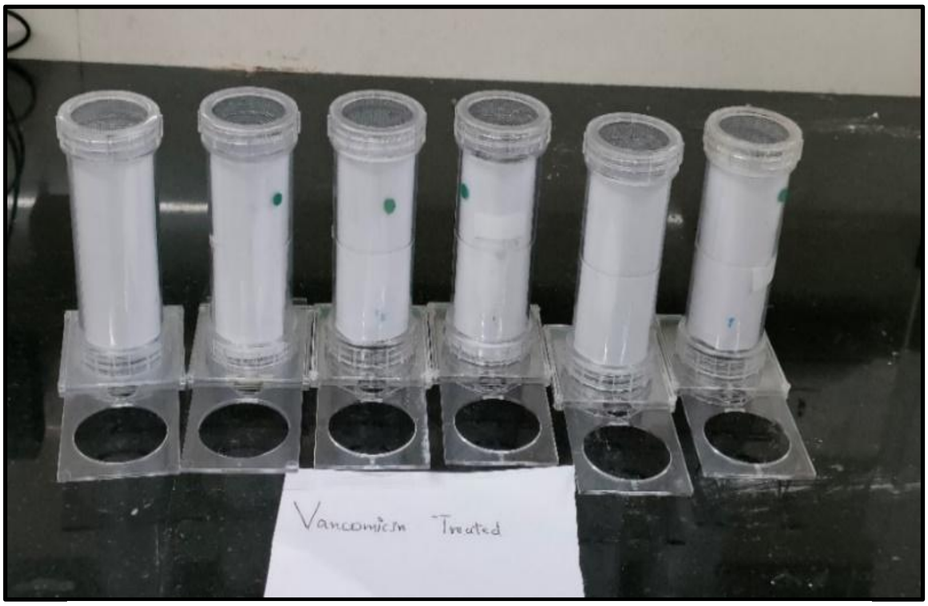

WHO tube test with Vancomycn treated population

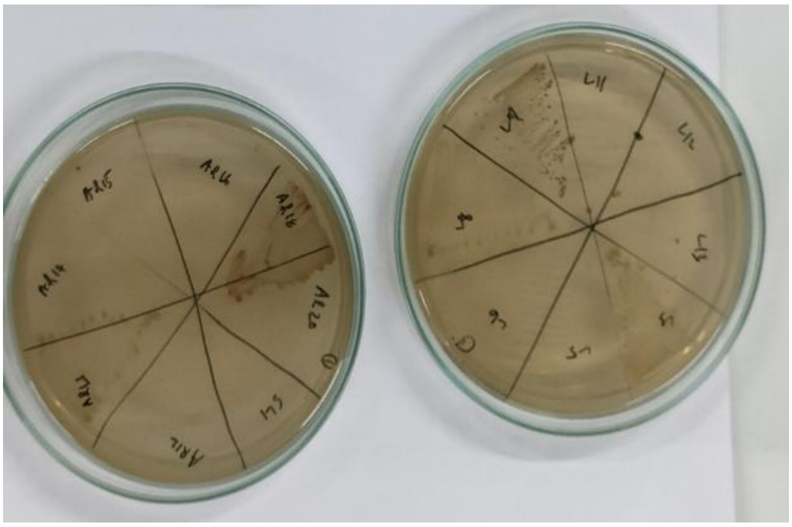

Supplementary Fig 8 -Growth of bacteria in Alphacypermethrin incorporated Minimal salt agar plates
