# Supplementary material for: Diversity of culturable gut bacteria and their role in conferring resistance to alpha-cypermethrin in field populations of Stegomyia aegypti
Source: Front Microbiol. 2026 Apr 14;17:1749347. doi: 10.3389/fmicb.2026.1749347 (PMC13121122; doi:10.3389/fmicb.2026.1749347)
Supplement: Supplementary file 3 [file Data_Sheet_3.PDF]

Phylogenetic trees constructed showed that the 16s rRNA sequences from the gut of *Stegomyia aegypti*, were found to cluster with *Acinetobacter baumannii*, *Shewanella decolorationis*, *Enterobacter mori*, *Microbacterium sp.* (Fig A,B,C,D). Sequences highlighted with red circles represent the 16s rRNA sequences isolated from the gut of *Stegomyia aegypti*. The scale indicates the number of substitutions per site.

A)

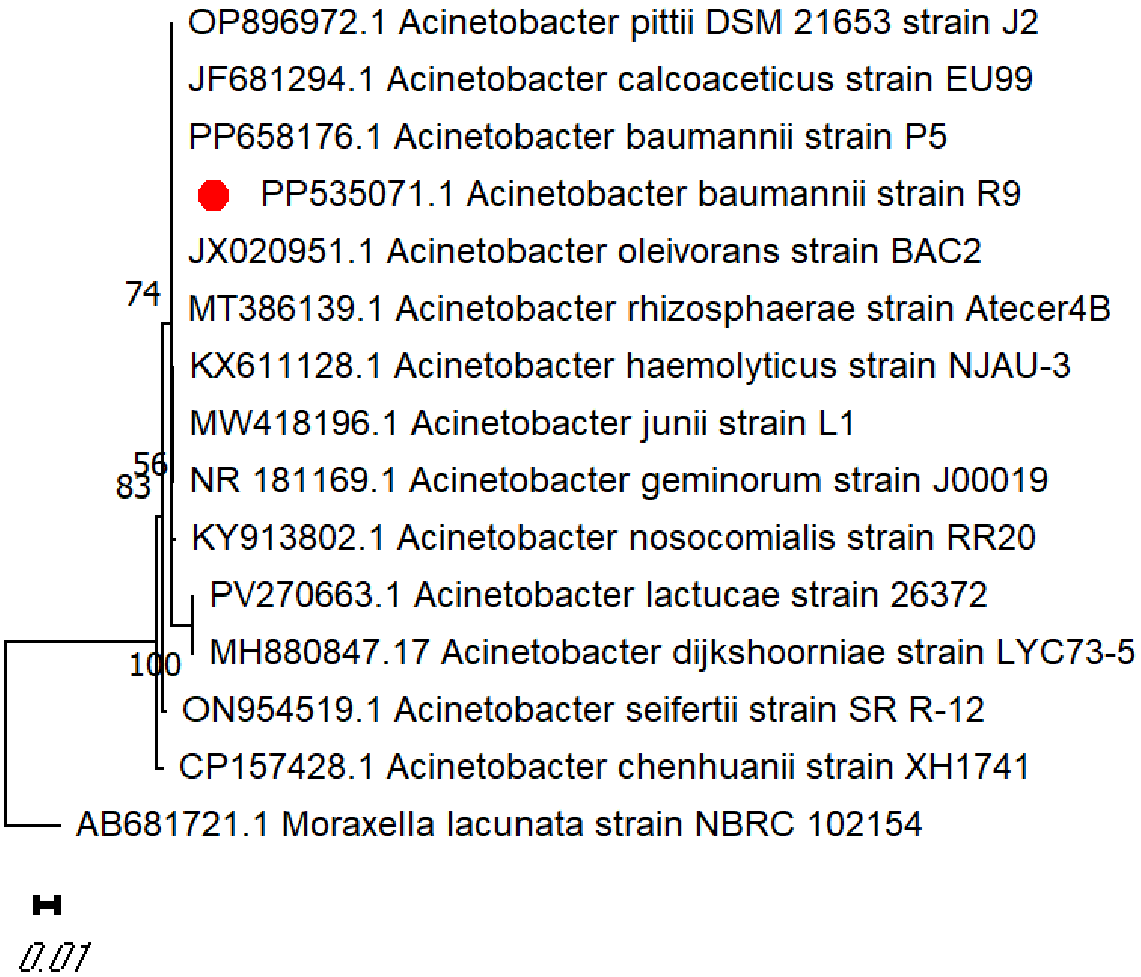

B)

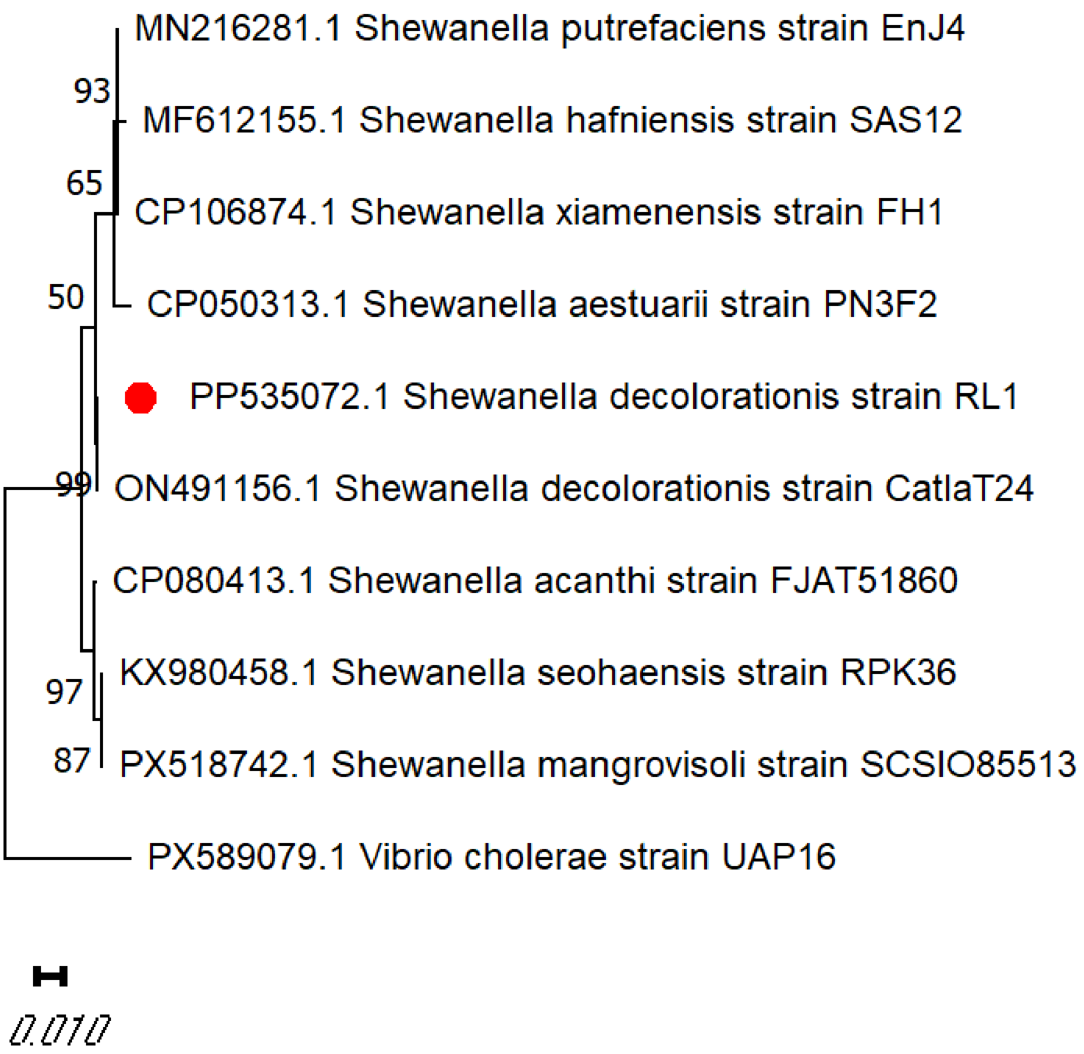
MN216281.1 Shewanella putrefaciens strain EnJ4
93
MF612155.1 Shewanella hafniensis strain SAS12
65
CP106874.1 Shewanella xiamenensis strain FH1
50
CP050313.1 Shewanella aestuarii strain PN3F2
PP535072.1 Shewanella decolorationis strain RL1
99
ON491156.1 Shewanella decolorationis strain CatlaT24
CP080413.1 Shewanella acanthi strain FJAT51860
KX980458.1 Shewanella seohaensis strain RPK36
97
87
PX518742.1 Shewanella mangrovisoli strain SCSIO85513
PX589079.1 Vibrio cholerae strain UAP16
0.010

C)

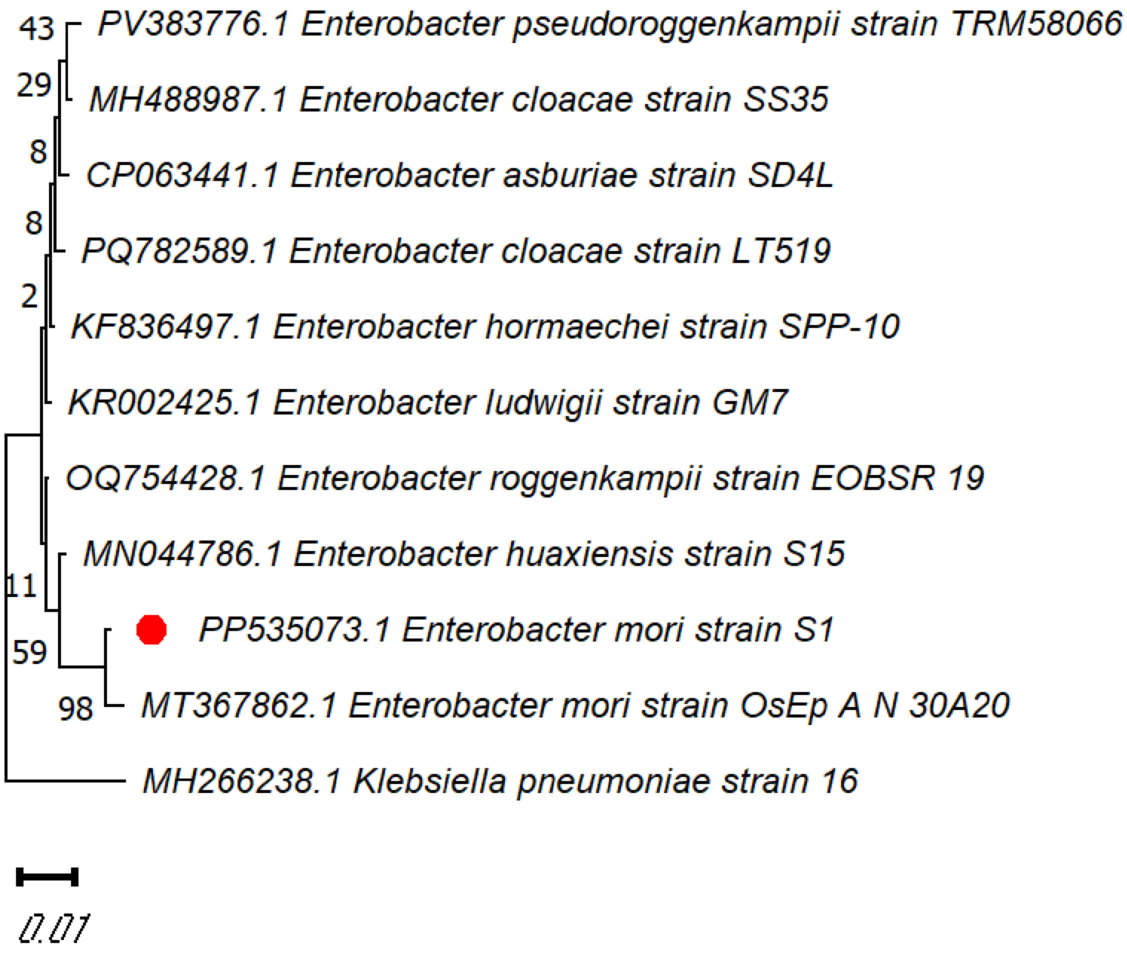

43
PV383776.1 Enterobacter pseudoroggenkampii strain TRM58066
29
MH488987.1 Enterobacter cloacae strain SS35
8
CP063441.1 Enterobacter asburiae strain SD4L
8
PQ782589.1 Enterobacter cloacae strain LT519
2
KF836497.1 Enterobacter hormaechei strain SPP-10
KR002425.1 Enterobacter ludwigii strain GM7
OQ754428.1 Enterobacter roggenkampii strain EOBSR 19
MN044786.1 Enterobacter huaxiensis strain S15
11
PP535073.1 Enterobacter mori strain S1
59
98
MT367862.1 Enterobacter mori strain OsEp A N 30A20
MH266238.1 Klebsiella pneumoniae strain 16
0.01

D)

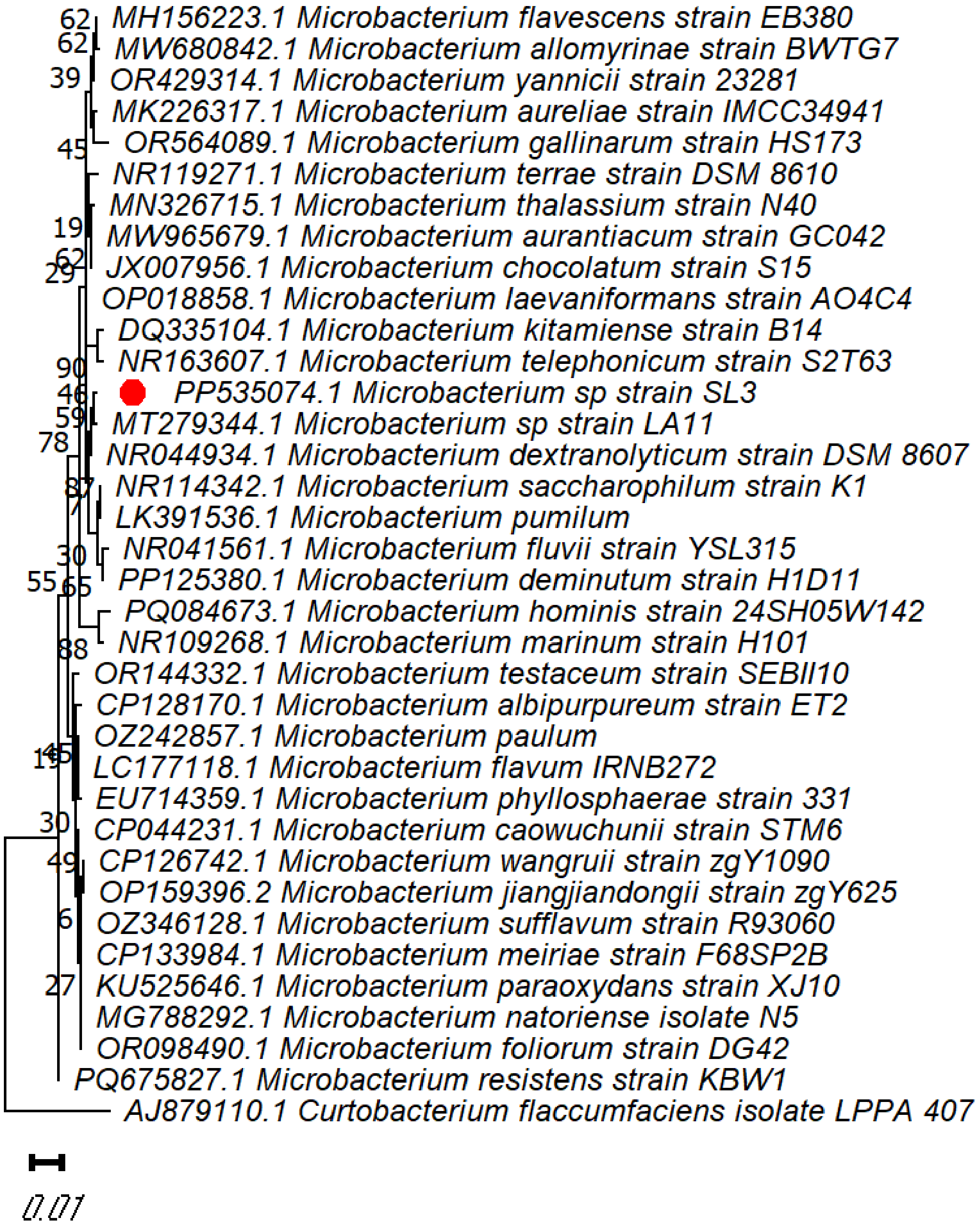

62
MH156223.1 Microbacterium flavescens strain EB380
62
MW680842.1 Microbacterium allomyrinae strain BWTG7
39
OR429314.1 Microbacterium yannicii strain 23281
MK226317.1 Microbacterium aureliae strain IMCC34941
45
OR564089.1 Microbacterium gallinarum strain HS173
NR119271.1 Microbacterium terrae strain DSM 8610
MN326715.1 Microbacterium thalassium strain N40
19
MW965679.1 Microbacterium aurantiacum strain GC042
62
29
JX007956.1 Microbacterium chocolatum strain S15
OP018858.1 Microbacterium laevaniformans strain AO4C4
DQ335104.1 Microbacterium kitamiense strain B14
NR163607.1 Microbacterium telephonicum strain S2T63
90
46
PP535074.1 Microbacterium sp strain SL3
59
MT279344.1 Microbacterium sp strain LA11
78
NR044934.1 Microbacterium dextranolyticum strain DSM 8607
87
NR114342.1 Microbacterium saccharophilum strain K1
7
LK391536.1 Microbacterium pumilum
30
NR041561.1 Microbacterium fluvii strain YSL315
55
65
PP125380.1 Microbacterium deminutum strain H1D11
PQ084673.1 Microbacterium hominis strain 24SH05W142
88
NR109268.1 Microbacterium marinum strain H101
OR144332.1 Microbacterium testaceum strain SEBII10
CP128170.1 Microbacterium albipurpureum strain ET2
OZ242857.1 Microbacterium paulum
15
45
LC177118.1 Microbacterium flavum IRNB272
EU714359.1 Microbacterium phyllosphaerae strain 331
30
CP044231.1 Microbacterium caowuchunii strain STM6
49
CP126742.1 Microbacterium wangruii strain zgY1090
OP159396.2 Microbacterium jiangjiandongii strain zgY625
6
OZ346128.1 Microbacterium sufflavum strain R93060
CP133984.1 Microbacterium meiriae strain F68SP2B
27
KU525646.1 Microbacterium paraoxydans strain XJ10
MG788292.1 Microbacterium natoriense isolate N5
OR098490.1 Microbacterium foliorum strain DG42
PQ675827.1 Microbacterium resistens strain KBW1
AJ879110.1 Curtobacterium flaccumfaciens isolate LPPA 407
0.01
