# Supplementary material for: Diversity of culturable gut bacteria and their role in conferring resistance to alpha-cypermethrin in field populations of Stegomyia aegypti
Source: Front Microbiol. 2026 Apr 14;17:1749347. doi: 10.3389/fmicb.2026.1749347 (PMC13121122; doi:10.3389/fmicb.2026.1749347)
Supplement: Supplementary file 4 [file Data_Sheet_4.PDF]

Supplementary Fig 6- MALDI-TOF Mass Spectra of bacterial isolates

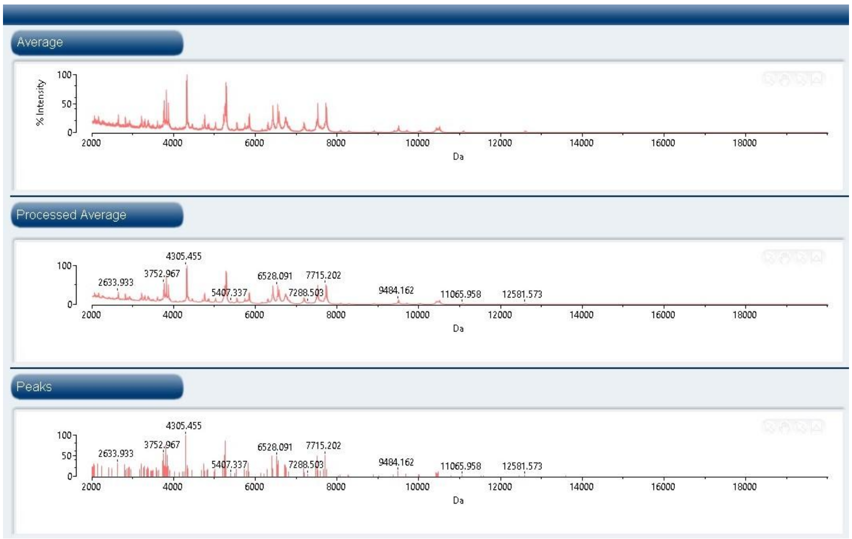

MALDI-TOF Mass Spectra of B.cereus group

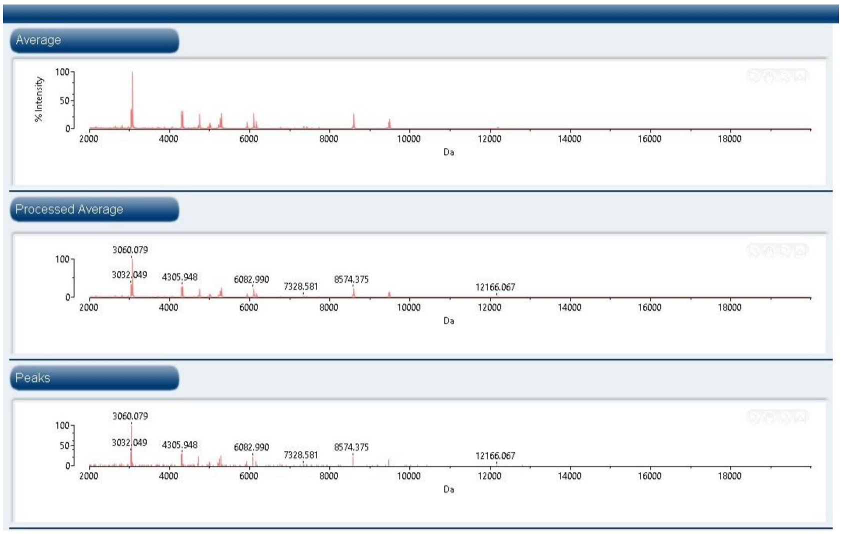

MALDI-TOF Mass Spectra of B.subtilis/ amyloliquefaciens/valismortis

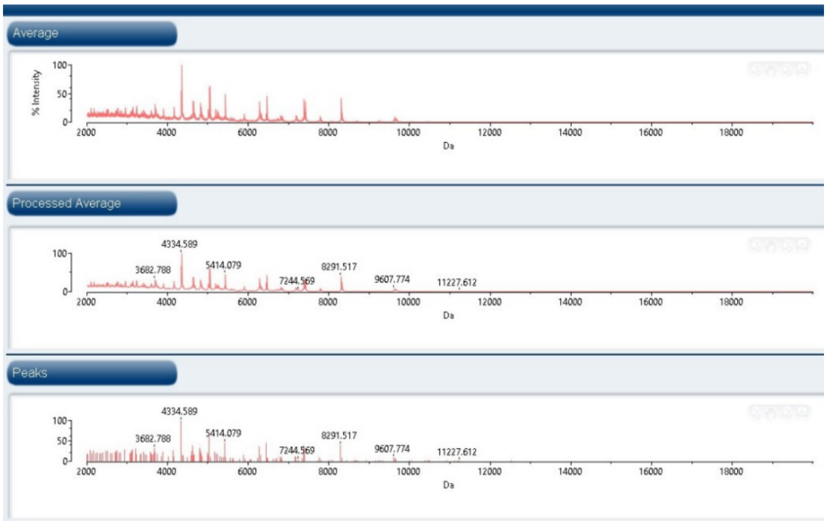

MALDI-TOF Mass Spectra of B.flexus

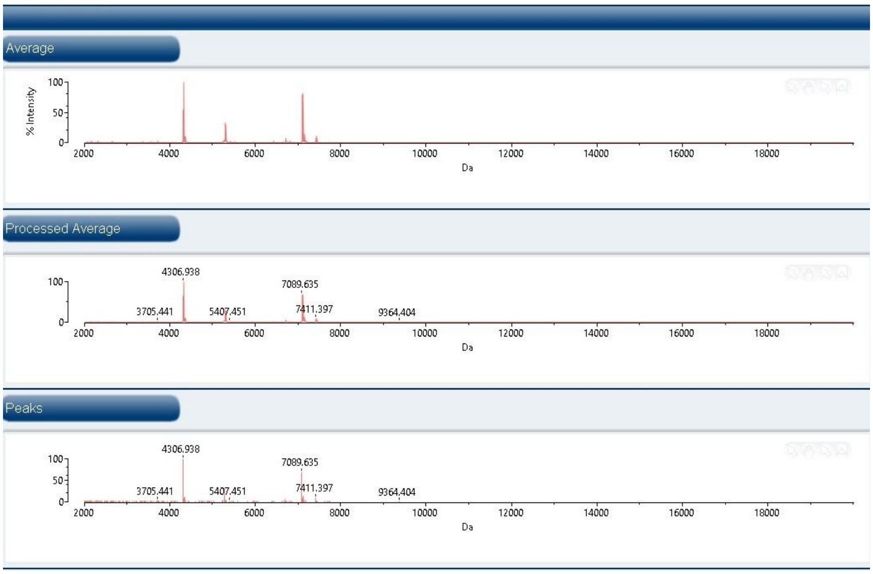

MALDI-TOF Mass Spectra of Bacillus altitudinis / pumilus

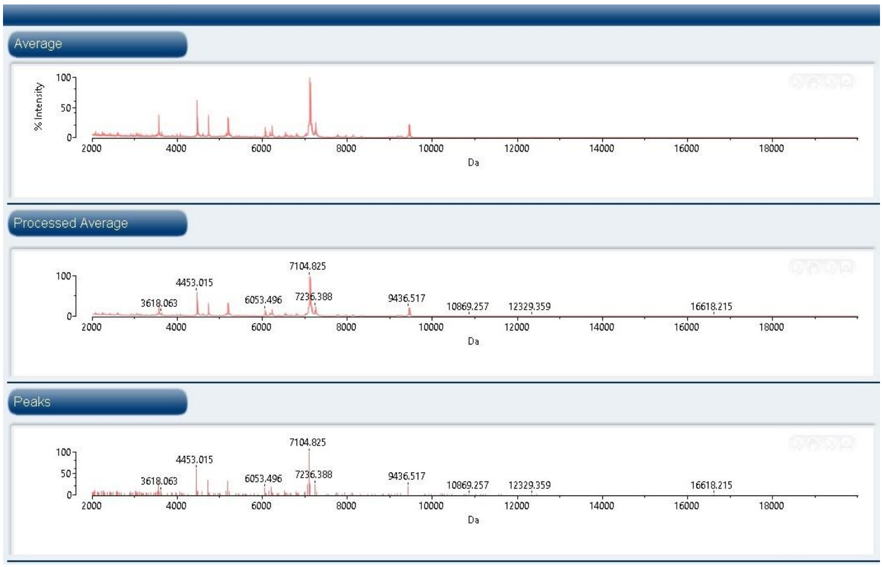

MALDI-TOF Mass Spectra of Lysinibacillus fusiformis

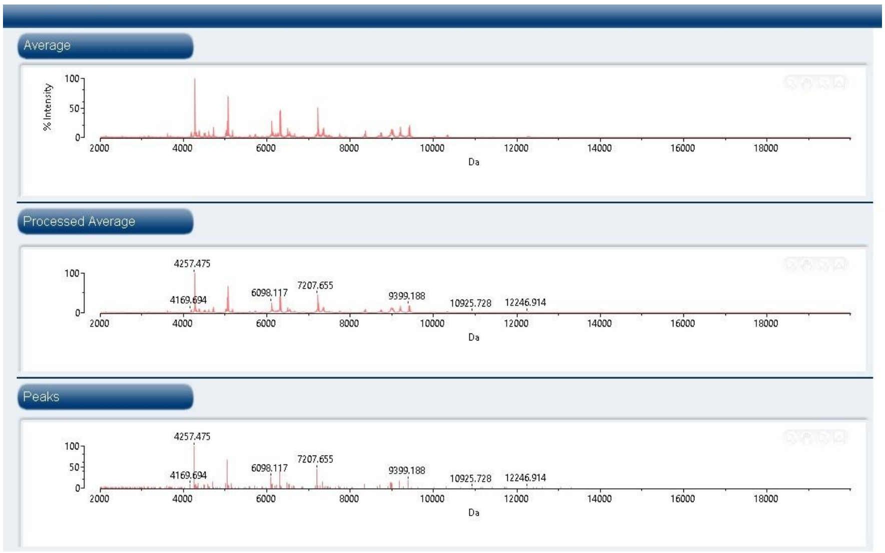

MALDI-TOF Mass Spectra of Lactobacillus garviae

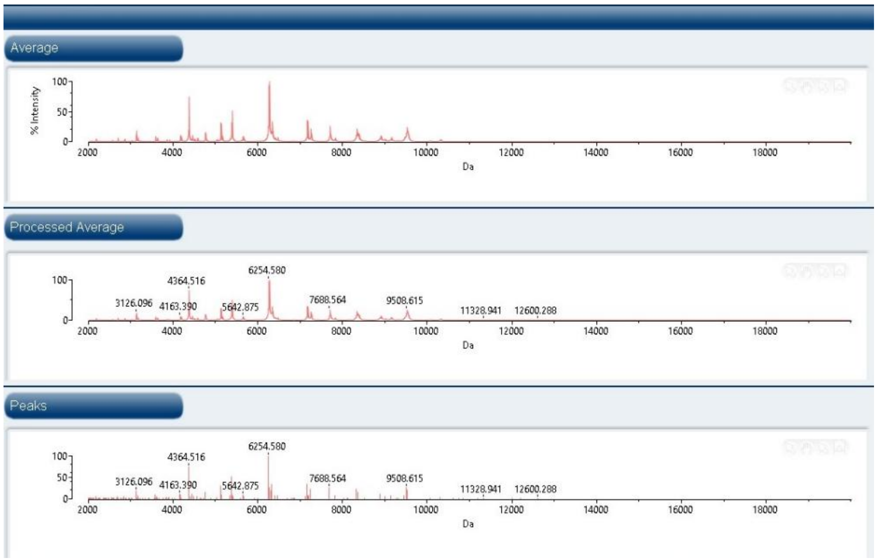

MALDI-TOF Mass Spectra of Enterobacter hormaechei

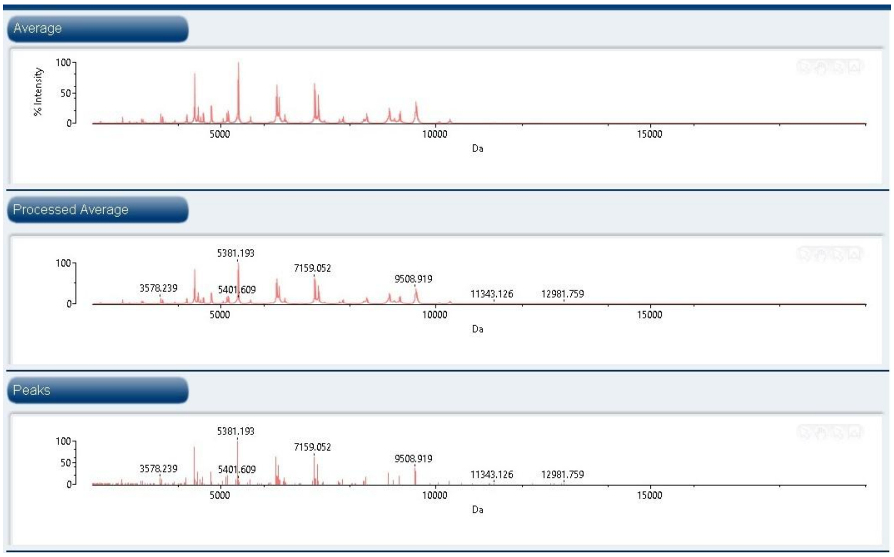

MALDI-TOF Mass Spectra of Enterobacter cloacae

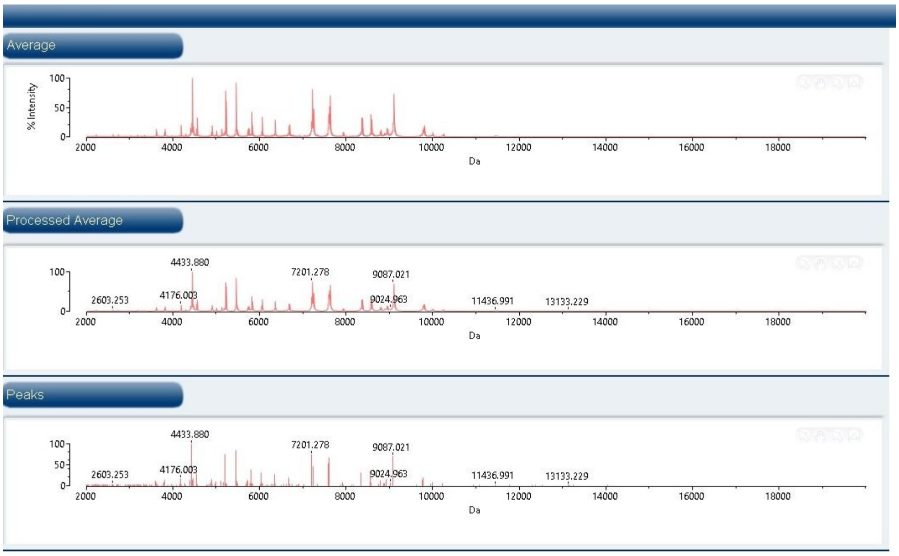

MALDI-TOF Mass Spectra of Pseudomonas aeruginosa

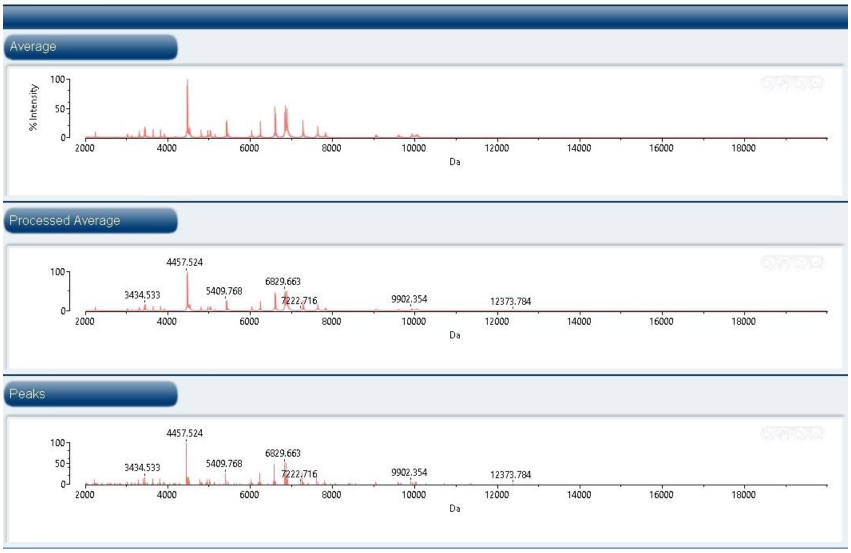

MALDI-TOF Mass Spectra of Chryseobacterium gleum

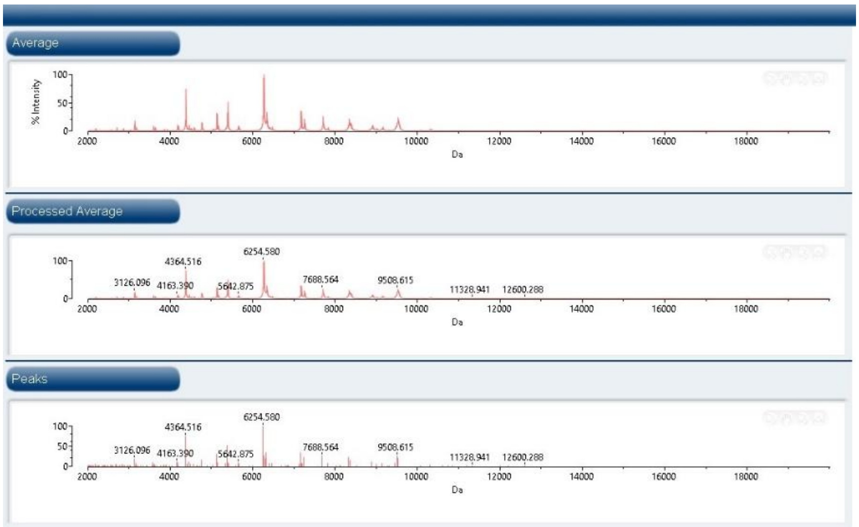

MALDI-TOF Mass Spectra of Staphylococcus aureus

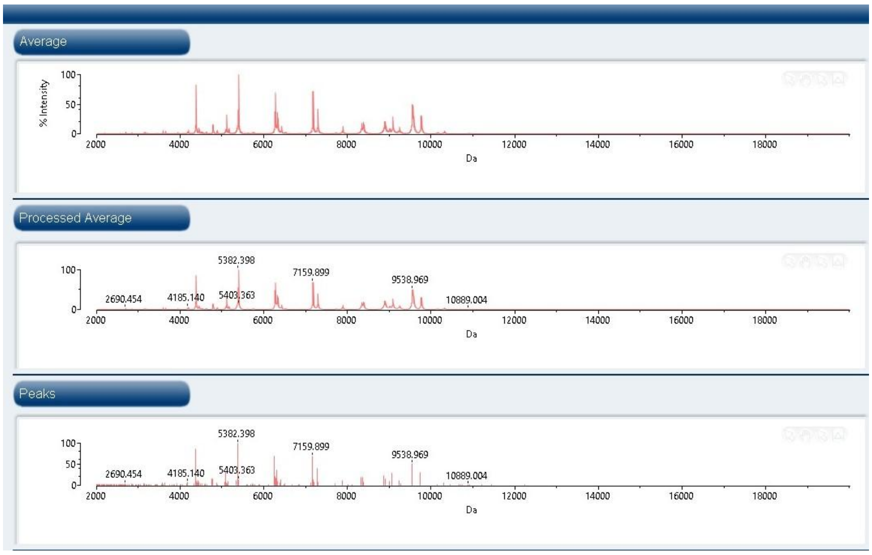

MALDI-TOF Mass Spectra of Staphylococcus epidermidis

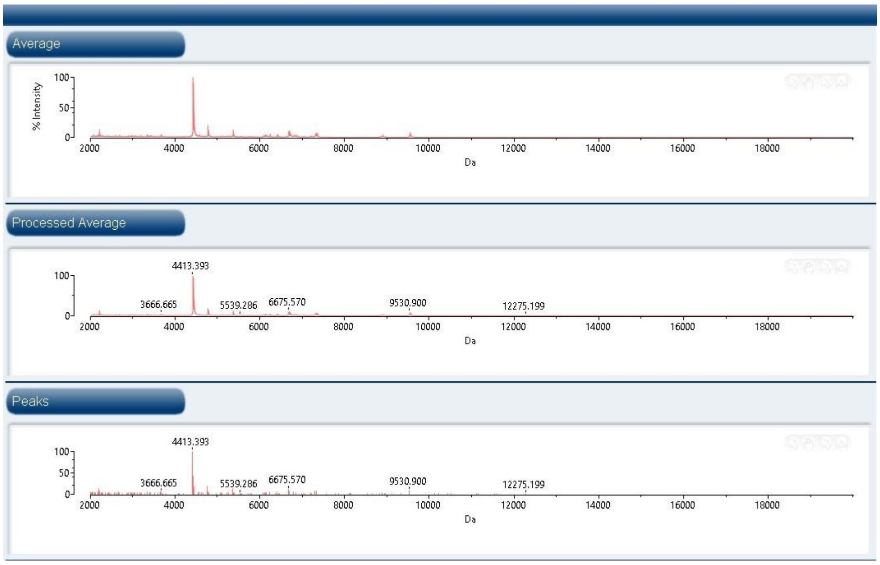

MALDI-TOF Mass Spectra of E.coli

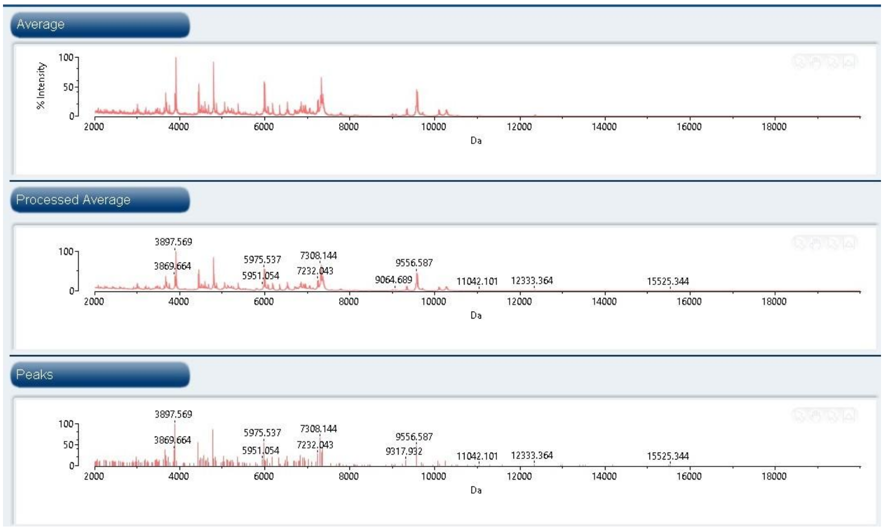

MALDI-TOF Mass Spectra of Enterococcus faecalis

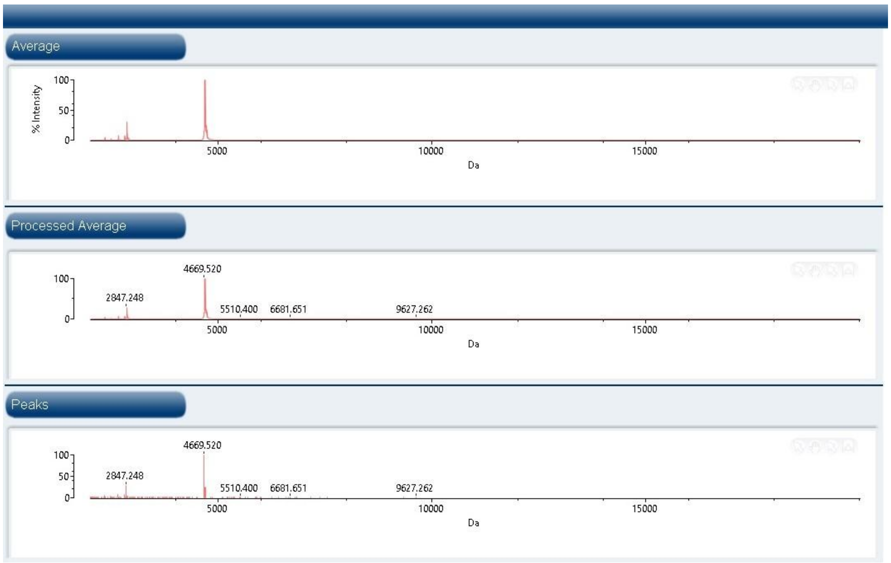

MALDI-TOF Mass Spectra of Enterococcus faecium

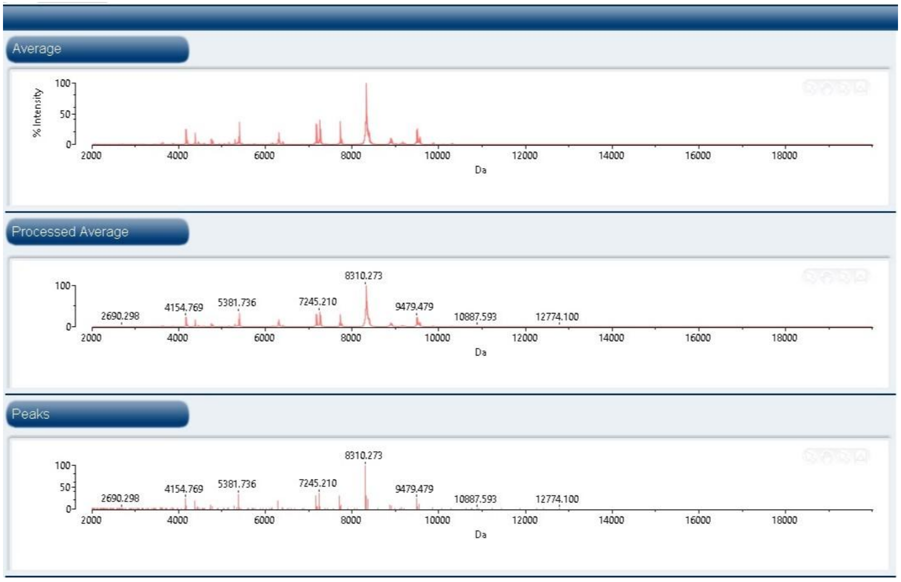

MALDI-TOF Mass Spectra of Klebsiella pneumoniae

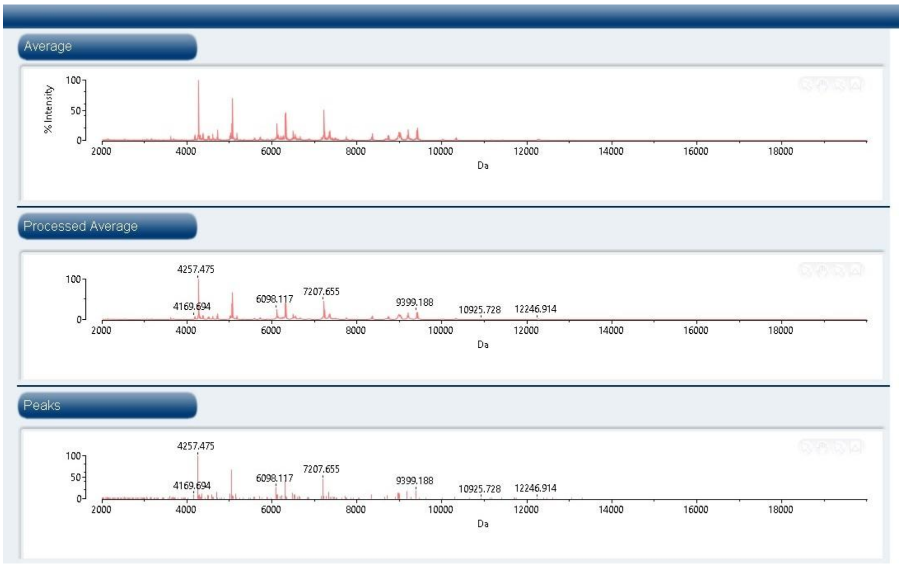

MALDI-TOF Mass Spectra of Aeromonas hydrophilia

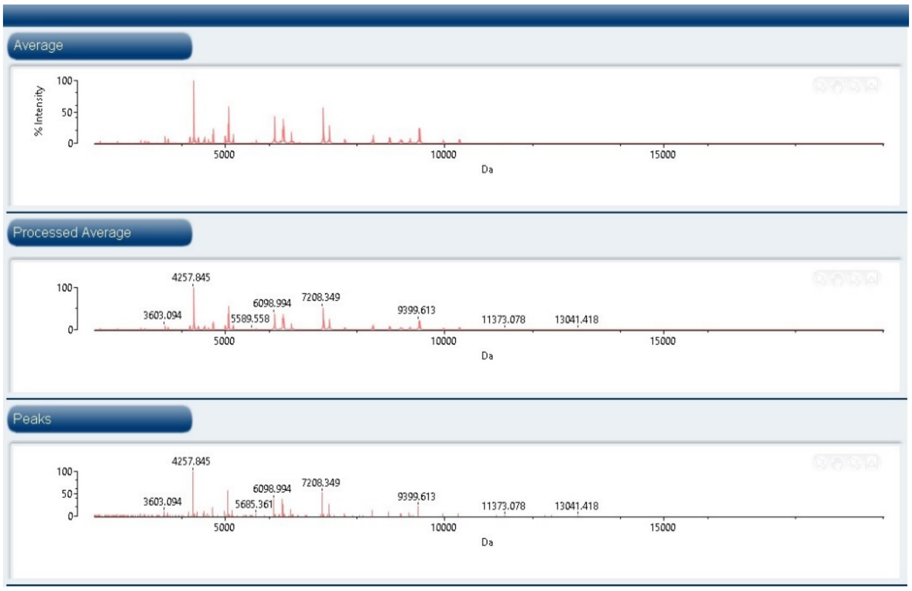

MALDI-TOF Mass Spectra of Aeromonas punctata
